# What is the role of microbial biotechnology and genetic engineering in medicine?

**DOI:** 10.1002/mbo3.1406

**Published:** 2024-03-31

**Authors:** Fernando Santos‐Beneit

**Affiliations:** ^1^ Institute of Sustainable Processes Valladolid Spain; ^2^ Department of Chemical Engineering and Environmental Technology, School of Industrial Engineering University of Valladolid Valladolid Spain

**Keywords:** antibiotics, biotechnology, cancer, genetic engineering, medicine, microbiology

## Abstract

Microbial products are essential for developing various therapeutic agents, including antibiotics, anticancer drugs, vaccines, and therapeutic enzymes. Genetic engineering techniques, functional genomics, and synthetic biology unlock previously uncharacterized natural products. This review highlights major advances in microbial biotechnology, focusing on gene‐based technologies for medical applications.

## Graphical abstract

Microbial biotechnology, the technological application of microorganisms, has been instrumental in producing significant natural bioactive products. These include antibiotics, antifungals, anticancer drugs, antiparasitics, antivirals, immunosuppressants, toxoid vaccines, and therapeutic enzymes. Certain microbial components have proven invaluable in the creation of genetic tools, such as CRISPR‐Cas systems and thermostable DNA polymerase enzymes. These tools are essential for the development of genetic engineering strategies. Genetic engineering, as a discipline, plays a crucial role in the rational and precise advancement of microbial biotechnology. Consequently, these two conceptual themes—microbial biotechnology and genetic engineering—exhibit a positive interplay. This review presents major advancements in microbial biotechnology, with a particular emphasis on gene‐based technologies within the medical field.

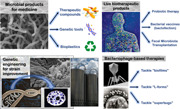

## 1 INTRODUCTION

For millennia, microorganisms have contributed to our daily lives by providing essentials like bread, beer, and wine. In recent times, the technological application of microorganisms—known as microbial biotechnology—has become a critical factor in producing vital natural bioactive compounds. These include antibiotics, antifungals, and anticancer agents. Moreover, the emergence of recombinant DNA technology owes much to microbial biotechnology, which has contributed with adequate enzymatic components (i.e., thermostable DNA polymerases and restriction enzymes, among others) and with extrachromosomal DNA structures (plasmids and cosmids) required for cloning and genetic modification of cells. Notably, the invention of the polymerase chain reaction (PCR)—where the Taq DNA polymerase (derived from the archaeal species *Thermus aquaticus*) amplifies DNA—has revolutionized molecular biology (Mullis & Faloona, 1987; Tindall & Kunkel, 1988). However, microbial biotechnology extends beyond alcohol fermentation, antibiotic synthesis, and molecular biology breakthroughs. It is a dynamic field where continuous exploration leads to fresh insights and discoveries.

**Figure 1:**
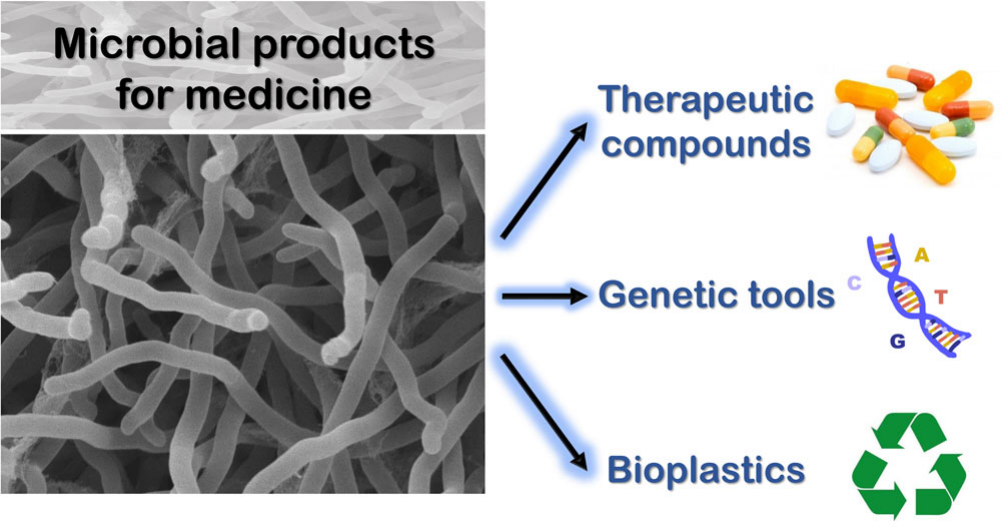
The utility of microbial products in medicine. Microbial products play a significant role in the development of various therapeutic agents, including antibiotics, antifungals, anticancer drugs, antiparasitics, antivirals, immunosuppressants, toxoid vaccines, and therapeutic enzymes. Certain microbial components are instrumental in creating genetic tools such as CRISPR‐Cas, thermostable DNA polymerase enzymes, and restriction‐modification systems. Additionally, some microorganisms possess the ability to produce biocompatible and biodegradable bioplastics, like polyhydroxyalkanoates, which are entirely synthesized by the microbial cell and can be used to manufacture medical devices.

The concept of genetic engineering, which involves the artificial manipulation, modification, and recombination of DNA or other nucleic acid molecules to alter organisms, has garnered significant interest over the past few decades. Recent advancements in genetic and molecular biology have propelled genetic engineering into the forefront of scientific and technological disciplines. Notably, two interconnected themes—microbial biotechnology and genetic engineering—exhibit positive feedback. Microbial biotechnology plays a pivotal role in shaping the field of genetic engineering. Simultaneously, genetic engineering contributes significantly to the precise development of microbial biotechnology. For instance, the discovery of clustered regularly interspaced short palindromic repeats (CRISPR)‐Cas components in bacteria has revolutionized genome editing. These breakthroughs can enhance the biotechnological capabilities of specific microorganisms, such as improving antibiotic production efficiency. These two disciplines are interdependent and often challenging to differentiate. Together, they have transformed both the industrial sector and the field of medicine. In medicine, microbial biotechnology and genetic engineering extend beyond therapeutic compound development (such as antibiotics and proteins). They also impact diagnosis, prevention, gene expression regulation, and the construction of medical devices using biocompatible biopolymers (summarized in Figure 1).

One of the landmark achievements in modern medicine was the sequencing of the human genome using classical DNA sequencing methods, which laid the foundation for studying human genomics. The Human Genome Project took a duration of 13 years (completed in 2003) and constituted the world's largest collaborative biological project in history. Over the last few decades, various whole genome sequencing platforms have been developed (i.e., Illumina, PacBio, Nanopore), enabling the sequencing of an immense number of genetic bases in a short period of time and inexpensively. These breakthroughs have deepened our understanding of the microbiota concept. Currently, we recognize that thousands of diverse microorganisms inhabit the human body—some with beneficial or neutral functions, while others have the potential to cause serious diseases (McCallum & Tropini, 2023). Consequently, understanding the genetic information of the microorganisms constituting our microbiota is crucial for discerning healthy and unhealthy states.

In medicine, microorganisms can be broadly categorized into five groups: viruses, bacteria, archaea, fungi, and parasites, each with its own complexity. The inappropriate use of antimicrobials (along with other factors) has led to the emergence of multidrug‐resistant microorganisms, commonly known as “superbugs.” These bacteria defy treatment with many available antibiotics, posing a major health challenge. (Crofts et al., 2017). As resistance to antibiotics increases, we may face a scenario akin to a “pre‐antibiotic” era (Makary et al., 2018). Additionally, despite advancements in drug design and manufacturing, cancer treatment still relies heavily on surgery, chemotherapy, and radiotherapy—often with severe side effects and limited curative outcomes. Immunotherapy offers promise for certain cancer types, but its applications remain limited in cases like pancreatic cancer and glioblastoma (Vivier et al., 2024). Therefore, to address infections and other diseases, including cancer, novel strategies must be developed and integrated into clinical practice. This review compiles major advances in microbial biotechnology with an historical perpective and with a special emphasis on gene‐based technologies, shaping the field of medicine.

## 2 THERAPEUTIC DRUGS OF MICROBIAL ORIGIN

Natural products have been utilized as traditional medicines to treat human diseases for thousands of years. In recent decades, if not the entire molecule, at least their molecular scaffolds have been employed to develop a variety of new therapeutic drugs (Miethke et al., 2021). Typically, these natural products are the result of the activation of secondary metabolism in microorganisms, particularly in bacteria and fungi (Martín et al., 2011; Santos‐Beneit, 2015; Santos‐Beneit et al., 2009). Chemotherapeutics derived from the secondary metabolism of microorganisms encompass antibiotics, antifungals, antivirals, anticancer, antiparasitic, and immunosuppressive agents, among others (Challis & Hopwood, 2003). The chemistry of these secondary metabolites is structurally diverse, based on several different backbone structures such as β‐lactams, polyketides, glycopeptides, and pyrroles (Davies, 2011). Among microbial secondary metabolites, antibiotics are the most commonly used compounds (Demain & Martens, 2017). The introduction of antibiotics into clinical practice revolutionized the treatment and management of diseases. Before the advent of these drugs, infectious diseases were the primary cause of morbidity and mortality in human populations.

### 2.1 Classical processes and procedures for drug discovery

The era of chemotherapy began at the dawn of the 20th century with the discovery of the first antibacterial compound of synthetic origin by German chemist Paul Ehrlich. This compound had an antagonistic effect against the spirochete that causes syphilis. The subsequent years saw the discovery of other compounds, both natural and synthetic, with antimicrobial activity such as penicillin, sulfanilamide, and streptomycin (Nicolaou & Rigol, 2018). Pharmaceutical companies then operated under the assumption that there was an inexhaustible supply of low‐molecular‐weight bioactive compounds in the biosphere. As a result, many other antibiotics were discovered in the following years. The general scheme followed by the pharmaceutical industry in the mid‐20th century for the discovery of natural products was based on two premises. First, the ability to isolate and grow a microorganism on common laboratory substrates, and second, the identification of its antibiotic production potential through biological antimicrobial tests, thereby selecting the best microbial isolates (Demain & Martens, 2017). Decades of searching for microbial products have led companies to generate vast collections of microorganisms that were isolated, characterized, and screened for bioactive compounds, primarily with antibiotic activity (Atanasov et al., 2021). However, it is now known that only a minimal percentage of microorganisms in the biosphere (~1%) can be cultured and isolated in laboratories (Ramírez‐Rendon et al., 2022). Consequently, classical processes have been replaced with more modern and rational methods to obtain new active compounds (see Section 2.2.2).

### 2.2 Antibiotics

In the 19th century, when Louis Pasteur and Robert Koch demonstrated that bacteria were the agents causing many infections, it was not yet known how these organisms (bacteria and other microorganisms) could be exploited to produce natural compounds capable of combating these bacterial infections, that is, antibiotics. It is important to distinguish between the terms “antibacterial” and “antibiotic.” While both antibiotics and antibacterials attack bacteria, these terms have different meanings. Antibacterials are agents that disinfect surfaces and eliminate potentially harmful bacteria (including soaps, detergents, skincare products, and household cleaners). However, unlike antibiotics, antibacterials are not used as medicines for humans or animals. In general, antibacterials can be classified as bacteriostats, sanitizers, disinfectants, and sterilizers based on their effectiveness in destroying microorganisms. Bacteriostats inhibit bacterial growth but do not kill bacteria. Sanitizers, on the other hand, kill a certain percentage of test microorganisms within a given period, while disinfectants destroy or irreversibly inactivate all test microorganisms (but not necessarily their spores). The most potent antibacterials, sterilizers, destroy all forms of bacteria, fungi, and other microorganisms (and their spores). Most of these antibacterial compounds (alcohols, aldehydes, peroxides, halogen‐releasing substances, anilides, biguanides, cresols, bisphenols, quaternary ammonium derivatives, heavy metals, ethylene oxide, and formaldehyde gases) are not directly produced by microorganisms (Maillard & Pascoe, 2024). Antibiotics, on the other hand, are often naturally produced by microorganisms or, in some cases, at least the backbone of the final antibiotic compound (i.e., semisynthetic antibiotics). However, several synthetic antibiotics are currently in the clinical pipeline and are widely used in clinics (Butler et al., 2023).

#### 2.2.1 Types of antibiotics

In general, it can be summarized that the primary types of antibiotics in use today were discovered during the first two‐thirds of the 20th century (Nicolaou & Rigol, 2018). However, to find the origins of the chemical industry that would lead to compounds with specific activity against particular organisms, we must trace back to the second half of the 19th century. This is what Paul Ehrlich defined as compounds that “exert their full action exclusively on the parasite harbored within the organism.” Leveraging the rise of the chemical industry at the beginning of the 20th century, a large‐scale, systematic screening program commenced in 1904 to find a specific drug for syphilis, a disease caused by the spirochete *Treponema pallidium* (Aminov, 2017). The outcome of this screening program was realized in 1907 with the development of an arsphenamine derivative. Years later, under the commercial name “Salvarsan,” it was proven effective in treating syphilis in humans (Nelson, 1910). This drug was in use until the introduction of penicillin in the 1940s. Penicillin was discovered by chance in 1928 by Alexander Fleming due to the accidental contamination of a culture plate by a fungus of the Penicillium family. By preparing a concentrate from a culture of this mold, Fleming demonstrated remarkable antibiotic activity against staphylococci. Following the discovery of penicillin, other compounds (natural or synthetic) were also discovered for the systematic treatment of infectious diseases. For instance, in 1935, the chemical dye Prontosil was shown to be curative in patients suffering from streptococcal infections. However, Prontosil itself was shown to be a precursor for the active drug. As later demonstrated, the dye was cleaved in the body to release p‐aminobenzenesulfonamide (sulfanilamide), which was responsible for the antibiotic activity (Aminov, 2017). Sulfonamides were inexpensive to produce and modify, opening a new era in medicine. Later, other antibiotic compounds produced by microorganisms, such as streptomycin and tetracycline, were discovered. In recent years, few new antibiotics have been developed for clinical use, with some exceptions being tigecycline, telithromycin, and daptomycin (Butler et al., 2023). Antibiotics can be classified based on various features. For example, according to their antimicrobial spectrum, they can be categorized as broad or limited‐spectrum antibiotics. A broad‐spectrum antibiotic destroys both Gram‐negative and Gram‐positive bacteria, while a limited‐spectrum antibiotic acts specifically against a type of microorganism or a specific group of microorganisms (Murray et al., 2021). Antibiotics can also be classified based on the cellular target they bind to and the cellular process they inhibit. Alternatively, antibiotics can be classified based on their type of synthesis (natural, synthetic, or semisynthetic), their structural class, and whether they have been approved for use in clinics (which varies depending on the regulatory agency) (Barberán et al., 2021). Table A1 shows the most important antibiotics in clinical use approved by major regulatory agencies, such as the US Food and Drug Administration (FDA), the European Medicines Agency (EMA), and other important national agencies, according to information collected from different databases (Drugs@FDA database, https://www.accessdata.fda.gov/scripts/cder/daf/; EUCAST, https://www.eucast.org/publications-and-documents/consultations; PRAC, 2021; STABILIS, https://www.stabilis.org/; CIMA, https://cima.aemps.es/cima/publico/home.html; AMMI Canada, https://choosingwiselycanada.org/infectious-disease). The main classes of antibiotics, according to their structures and the cellular processes they inhibit, are described below.

##### Inhibition of cell wall synthesis and/or cell membrane integrity

The most common mechanism of antibiotic activity is the interference with bacterial cell wall synthesis (Butler et al., 2023). Peptidoglycan, the major structural component of bacterial cell walls, consists of layers of alternating molecules of N‐acetylglucosamine and N‐acetylmuramic acid cross‐linked with peptide bridges. This creates a rigid mesh coating for the bacteria. Most of the cell wall‐active antibiotics belong to the β‐lactam antibiotics group (i.e., penicillins, cephalosporins, cephamycins, carbapenems, monobactams), which share a common β‐lactam ring structure. These antibiotics target specific enzymes (i.e., transpeptidases, transglycosylases, and carboxypeptidases) responsible for the construction of peptidoglycan, collectively known as penicillin‐binding proteins (PBPs) (Aminov, 2017). Bacteria can produce β‐lactamases that inactivate the β‐lactam antibiotics. Interestingly, the β‐lactamases belong to the same family of serine proteases as the PBPs. Many different β‐lactamases have been described, some showing a broad range of activity for penicillins, cephalosporins, or carbapenems, and others being specific for a certain type of β‐lactam antibiotic. For this reason, β‐lactam antibiotics are often combined with β‐lactamase inhibitors in the clinic. The β‐lactamase inhibitors (i.e., clavulanic acid, sulbactam, tazobactam, avibactam) are relatively inactive by themselves, but when combined with some β‐lactam antibiotics, they are quite effective in treating infections caused by β‐lactamase‐producing bacteria (Kumar et al., 2023). Among the β‐lactam antibiotics, penicillins are highly effective antibiotics with extremely low toxicity. However, many pathogens have developed resistance against them since their introduction in clinics. Cephalosporins and cephamycins, which exhibit the same mechanism of action as penicillins, have improved pharmacokinetic properties (such as a longer half‐life) and enhanced activity against a wide range of bacterial species. However, resistance to most cephalosporins and cephamycins has also been developed. Other classes of β‐lactam antibiotics are carbapenems and monobactams. Carbapenems (such as imipenem or meropenem) are widely prescribed broad‐spectrum antibiotics that are active against many groups of organisms. In contrast, monobactams (such as aztreonam) are narrow‐spectrum antibiotics that are active only against a specific group of bacteria (aerobic Gram‐negative bacteria) (Murray et al., 2021).

Glycopeptides, lipopeptides, and polypeptides are other classes of antibiotics that act against the synthesis of the bacterial cell wall. Glycopeptides are complex structures consisting of a peptide core and sugar molecules attached to the aglycone component at various sites (Butler et al., 2023). Vancomycin and teicoplanin are the most widely recognized members of the large family of glycopeptide antibiotics (Santos‐Beneit et al., 2014). Both antibiotics interact with the D‐alanine‐D‐alanine termini of the pentapeptide side chains of the peptidoglycan. Some organisms are intrinsically resistant to vancomycin and teicoplanin because the pentapeptide of their cell walls terminates in D‐alanine‐D‐lactate, which does not bind these antibiotics (Santos‐Beneit & Martín, 2013). Other mechanisms of resistance to glycopeptides have also been identified (Santos‐Beneit, 2021). However, the onset of resistance to glycopeptides in major pathogens has been delayed compared to β‐lactam antibiotics (Santos‐Beneit, Ordóñez‐Robles, et al., 2017). Lipopeptides are amphiphilic molecules containing a short linear or cyclic oligopeptide (polar moiety) and a linear or branched fatty acid of varying lengths (apolar moiety) (Hervin et al., 2023). Daptomycin, approved for clinical use at the beginning of the 21st century, has a distinct mechanism of action compared to β‐lactam antibiotics or glycopeptide antibiotics, disrupting multiple aspects of bacterial cell membrane function (Butler et al., 2023). Daptomycin has potent activity against Gram‐positive bacteria, but not against Gram‐negative bacteria, due to the different composition and permeability of the cell envelope (cell membranes and cell wall) of these two groups of bacteria (Murray et al., 2021). Polypeptide antibiotics are a chemically diverse class of compounds containing nonprotein polypeptide chains. The most important examples of this class of antibiotics include bacitracin, colistin, and polymyxin B (Aminov, 2017). Bacitracin inhibits bacterial viability by hampering the movement of the peptidoglycan precursors through the cytoplasmic membrane to the cell wall, damaging the bacterial cytoplasmic membrane, and inhibiting the process of transcription. On the other hand, polymyxin B and E (colistin) insert into bacterial membranes like detergents by interacting with the phospholipids of the membrane, producing increased cell permeability and eventual cell death (Murray et al., 2021). Finally, other non‐β‐lactam antibiotics targeting the cell envelope of bacterial cells include isoniazid, ethionamide, ethambutol, and cycloserine, which are used for the treatment of mycobacterial infections (i.e., mycobacteria have a unique cell envelope composition among all bacterial species that offers alternative targets for distinct compounds) (Butler et al., 2023).

##### Inhibition of protein synthesis

The second‐largest class of antibiotics are those capable of inhibiting protein synthesis in bacteria. Among these antibiotics, the most important classes include aminoglycosides, tetracyclines, macrolides, ketolides, glycylcyclines, streptogramins, oxazolidinones, and lincosamides (Aminov, 2017). Aminoglycosides, such as streptomycin, kanamycin, gentamicin, tobramycin, and amikacin, consist of amino sugars linked through glycosidic bonds to an aminocyclitol ring. These antibiotics function by causing the premature release of peptide chains from the 30S ribosome, thereby specifically inhibiting bacterial protein synthesis. Among these aminoglycosides, amikacin is the most active and commonly used antibiotic, primarily used to treat infections with Gram‐negative rods. Tetracyclines, such as tetracycline, doxycycline, and minocycline, prevent polypeptide elongation by blocking the binding of aminoacyl‐transfer RNA (tRNA) to the 30S ribosome–mRNA complex (Murray et al., 2021). All tetracyclines have a similar spectrum of activity and show broad‐spectrum activity against Gram‐positive and some Gram‐negative bacteria, such as *Neisseria* or some Enterobacteriaceae (Waitayangkoon et al., 2023). Macrolides, such as erythromycin, azithromycin, clarithromycin, and roxithromycin, prevent polypeptide elongation at the 50S ribosome by binding to the 23S ribosomal RNA (rRNA) (Murray et al., 2021). The model macrolide antibiotic, erythromycin, has a basic structure consisting of a macrocyclic lactone ring bound to two sugars, desosamine and cladinose (Murray et al., 2021). Modification of the macrolide structure led to the development of azithromycin, clarithromycin, and roxithromycin (Butler et al., 2023). A similar mode of action to that of macrolides is performed by ketolides, streptogramins, and lincosamides (Waitayangkoon et al., 2023). Finally, two distinct antibiotics that inhibit protein synthesis, being the only members approved for clinical use within their corresponding class, are tigecycline and linezolid (Butler et al., 2023). Tigecycline is the first representative of a new class of antibiotics named glycylcyclines, which inhibit protein synthesis in the same manner as the tetracyclines but with a higher binding affinity for the ribosome and with less affectation by efflux or enzymatic modification. Linezolid, the only antibiotic of the oxazolidinone class currently in clinical use, prevents the initiation of protein synthesis at the 50S ribosome with a unique mechanism that distorts the binding site for tRNA, inhibiting the formation of the 70S initiation complex. Because of its importance, this antibiotic is reserved as a last‐resort treatment for difficult infections caused by multidrug‐resistant bacteria (Murray et al., 2021).

##### Inhibition of nucleic acid synthesis

The third group of most important antibiotics in clinical use today are those that inhibit nucleic acid synthesis. Among this group, a major class is constituted by quinolones, which inhibit DNA replication by binding to topoisomerase type II (DNA gyrase) or topoisomerase type IV (Butler et al., 2023). The first quinolone used in clinical practice was nalidixic acid, which was used to treat urinary tract infections. However, this antibiotic later fell out of use (Aminov, 2017). Currently, this drug has been replaced by newer and more active quinolones, such as ciprofloxacin, levofloxacin, and moxifloxacin (Murray et al., 2021). These antibiotics have excellent activity against Gram‐positive and Gram‐negative bacteria, although resistance can develop quickly (Nicolaou & Rigol, 2018). Other antibiotics that block DNA synthesis include metronidazole, which disrupts bacterial DNA by specifically hampering the action of the bacterial nitroreductase enzyme, leading to the production of cytotoxic compounds that disrupt the bacterial DNA, and clofazimine, which binds to guanine bases of mycobacterial DNA, thereby blocking the template function of the DNA and inhibiting bacterial replication (Aminov, 2017). Another important class of antibiotics capable of inhibiting nucleic acid synthesis is constituted by rifamycins, which prevent DNA transcription by binding and blocking the RNA polymerase of bacteria (Butler et al., 2023). The rifamycin group includes the classic rifamycin drugs, such as rifampin or rifampicin, as well as the derivatives rifabutin, rifapentine, rifalazil, and rifaximin (Bobba & Khader, 2023). Rifamycins are particularly effective against mycobacteria and are therefore used to treat tuberculosis and leprosy infections, caused by *Mycobacterium tuberculosis* and *Mycobacterium leprae*, respectively. Specifically, rifampicin is used for the treatment of tuberculosis in combination with other antibiotics, such as pyrazinamide, isoniazid, and ethambutol (Murray et al., 2021). For *Mycobacterium leprae* infections (leprosy), rifampicin is normally used together with clofazimine (sold under the brand name “Lamprene”) and dapsone, which inhibits dihydropteroate synthase (Le et al., 2023). Dapsone, along with sulfonamides and trimethoprim, is grouped within the antibiotics known as antimetabolites (Murray et al., 2021). Antimetabolites act by mimicking purines and pyrimidines that are required for DNA synthesis or by interfering with the native synthesis of nucleotides (Butler et al., 2023). Sulfonamides, for example, are antimetabolites that compete with p‐aminobenzoic acid, an intermediate in the synthesis of folic acid, thereby preventing the synthesis of folic acid, which is required by certain microorganisms to produce the precursors for nucleotide synthesis (Aminov, 2017). Trimethoprim, similar to sulfonamides, inhibits dihydrofolate reductase and disrupts folic acid synthesis (Murray et al., 2021). Trimethoprim is commonly combined with sulfamethoxazole, a sulfonamide antibiotic, to produce a synergistic effect in the inhibition of folic acid synthesis. The trimethoprim‐sulfamethoxazole combination therapy, known as cotrimoxazole or bactrim among other names, is effective against a large variety of Gram‐positive and Gram‐negative microorganisms and is the therapy of choice for the treatment of acute and chronic urinary tract infections (Batra et al., 2017).

##### Inhibition of other essential cell processes of bacteria

There are antibiotics currently under development, in clinical trials, or part of a fast‐track accelerated approval process, that target essential components for bacterial cell viability, distinct from those previously mentioned. For instance, bedaquiline, an antibiotic used to treat multi‐drug‐resistant tuberculosis, inhibits the proton pump for ATP synthase in mycobacteria. ATP production is crucial for cellular energy production. Bedaquiline is the inaugural member of a new class of drugs known as diarylquinolines (Worley & Estrada, 2014). The specific component of ATP synthase that bedaquiline affects is subunit C, encoded by the gene atpE. Consequently, mutations in atpE can lead to antibiotic resistance (Worley & Estrada, 2014). Another example is the essential bacterial cell division protein FtsZ, which is emerging as a promising new antibiotic target (Andreu et al., 2022). This protein is vital for the successful completion of the bacterial cell division process and is responsible for dividing the parental bacterial cell into two daughter cells in most bacteria (Santos‐Beneit, Roberts, et al., 2017). Therefore, inhibiting this protein prevents bacterial proliferation. Several natural, semisynthetic, and synthetic FtsZ inhibitors have already been discovered and tested (Kifayat et al., 2023). Among these inhibitors, benzodioxanes and benzamides have shown the most promising results against both Gram‐positive and Gram‐negative bacteria. Several candidates could become available in clinics in the coming years (Suigo et al., 2023). Lastly, a recent publication proposed the LptB2FGC protein complex as a novel target to combat carbapenem‐resistant *Acinetobacter baumannii* (CRAB) infections, which currently have very limited treatment options in hospitals. Zosurabalpin, a clinical candidate derived from the macrocyclic peptide class of antibiotics, has been shown to inhibit this novel target in CRAB. It blocks the transport of an essential bacterial lipopolysaccharide from the inner membrane to its destination on the outer membrane. Without the outer membrane, the Gram‐negative *A. baumannii* bacterium is less likely to survive and becomes vulnerable to other antibiotics that could be combined with zosurabalpin to treat CRAB infections. This promising antibiotic has already been effectively tested to treat highly drug‐resistant CRAB isolates both in vitro and in mouse infection models, overcoming existing antibiotic resistance mechanisms (Zampaloni et al., 2024). In summary, despite the existing antibiotics in the clinical pipeline, there is a need for new classes of antibiotics that inhibit previously undrugged targets to overcome the current resistance mechanisms developed by pathogenic bacteria.

#### 2.2.2 Genetic engineering for enhancing antibiotic production and creating diversity

**Figure 2:**
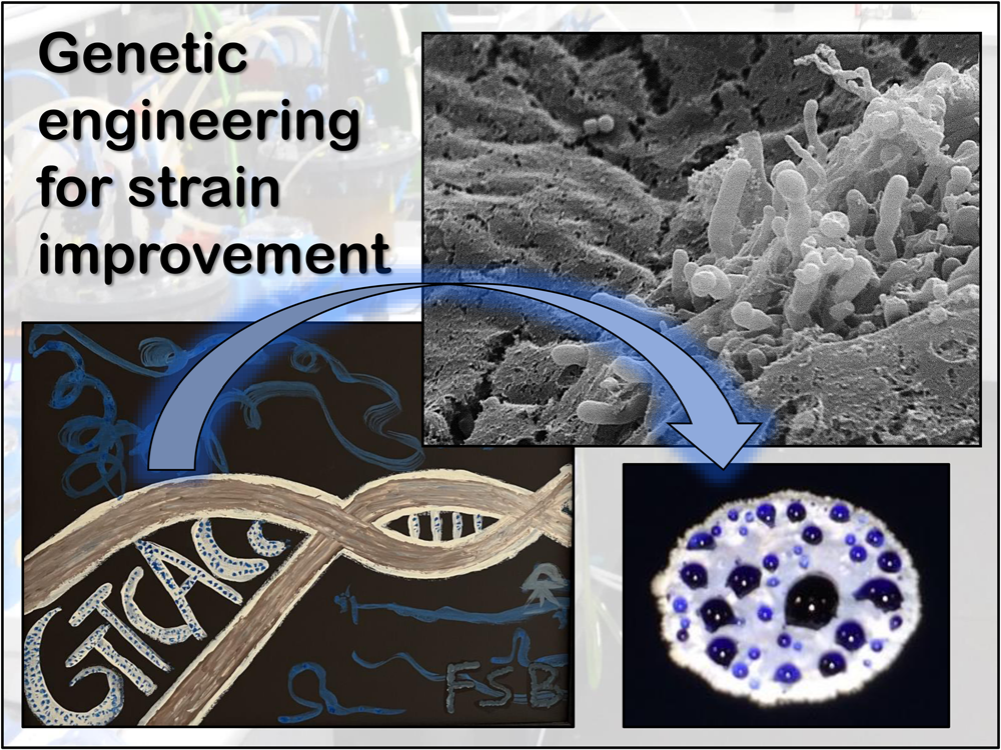
The role of genetic engineering in strain improvement. Modifying a specific DNA sequence (even a single nucleotide) can cause a complete change in the bacteria's phenotype, leading to the overproduction of a certain type of antibiotic. The figure illustrates this with an example of soil bacteria *Streptomyces*. The cells are shown just as they emerge from the solid substrate. The figure also depicts the overproduction of a pigmented antibiotic, actinorhodin, by a colony of the species *Streptomyces coelicolor*. Streptomycetes are responsible for producing most of the naturally occurring antibiotics in use today.

The identification of microorganisms capable of producing useful therapeutic agents is a crucial step for the pharmaceutical industry. However, the need for efficient large‐scale production is equally critical. For many years, pharmaceutical companies have had to induce genetic mutations in antibiotic‐producing strains to generate genetic diversity. Simultaneously, they adjusted the composition of the bacterial growth media based on the specific effects of these genetic mutations. In the second half of the 20th century, pharmaceutical companies employed classical methods to improve antibiotic production yields or generate new derivatives. These methods were based on generating random mutations, either through ultraviolet radiation or using mutagenic chemical agents, followed by a screening process. This process proved quite effective, allowing production titles of g/l for most commercial compounds (Demain & Martens, 2017). Even a single nucleotide mutation can cause a significant change in a cell's phenotype. For instance, a point mutation in a specific regulator can increase antibiotic yields hundreds of times higher than without that given mutation or enhance a bacterium's resistance to a specific antibiotic (Fernández‐Martínez et al., 2012; Martín‐Martín et al., 2017; Santos‐Beneit, 2018; Santos‐Beneit et al., 2011). Therefore, the ability to generate mutant bacterial strains, either through genetic engineering approaches or randomly, has undoubtedly changed the paradigm of antibiotic production processes (Figure 2).

In recent decades, the advent of modern genetic engineering techniques has led most pharmaceutical companies to completely overhaul their antibiotic discovery programs. For instance, the rearrangement of modular polyketide synthase genes in combinatorial polyketide biosynthesis has resulted in the creation of “unnatural” natural products that did not previously exist in nature (Menzella et al., 2005). This type of combinatorial genetic engineering strategy falls under a broader discipline known as synthetic biology, which merges microbiology, biotechnology, and genetic engineering to design and construct new biological compounds. Many of these synthetic biology approaches have been applied to type I and type II polyketides (Yuzawa et al., 2018). Type I and type II polyketides consist of carbon skeletons, aromatic or otherwise, which are further modified by tailoring enzymes to produce the final bioactive compounds. The carbon skeleton comprises β‐keto groups with varying degrees of reduction, produced by a series of Claisen condensation reactions of short‐chain acyl‐CoA molecules (such as acetyl‐CoA or malonyl‐CoA) through the activity of specific enzymes known as polyketide synthases (PKSs) (Malico et al., 2020). Therefore, the final structure of the compound can be modified either by altering the tailoring reactions (i.e., methylation, glycosylation, oxygenation) or by combining the PKSs that form the carbon skeleton. The generation of new polyketide structures can enhance the properties of the original compounds or create new chemical structures with novel activities. From a medical and industrial perspective, these strategies are of great importance since polyketides represent the largest class of natural products that have found applications in medicine, agriculture, and animal health. Examples of FDA‐approved polyketides include antibiotics (i.e., rifamycin), antifungal agents (i.e., amphotericin), immunosuppressive agents (i.e., tacrolimus), anticancer drugs (i.e., geldanamycin), cholesterol‐lowering agents (i.e., lovastatin), anthelmintic agents (i.e., avermectin), insecticides (i.e., spinosad), and growth‐promoting factors for ruminants (i.e., monensin) (Yang et al., 2023).

On the other hand, recent advances in the fields of genomics, transcriptomics, proteomics, and metabolomics have enabled the activation of previously silenced cryptic antibiotic biosynthetic pathways in the producing strain, thereby increasing the number of promising biosynthetic pathways for new antibiotic screening. Specifically, the information obtained from transcriptomic studies allows for the adjustment of appropriate regulatory networks to activate or increase the expression of a specific antibiotic gene cluster. Alternatively, heterologous expression represents a major approach for activating these silent gene clusters. In addition, recent advances in the field of metagenomics have been instrumental in identifying new gene clusters from uncultured communities capable of producing new antibiotics. Metagenomics involves the direct study of a set of genomes from a specific environment, using samples from it, without the need to isolate and grow the organisms being analyzed. However, it does require the use of appropriate computer systems (Ustick et al., 2021). In this context, bioinformatics has become an essential tool for conducting these types of analyses, underscoring the importance of this discipline for obtaining new bioactive compounds, and therefore, for the pharmaceutical industry and medical fields. Specific software and bioinformatics tools have been developed for the rapid identification of genes of interest from genomic data. For instance, the antiSMASH online platform enables the rapid genome‐wide identification, annotation, and analysis of secondary metabolite biosynthesis gene clusters in bacterial and fungal genomes (Blin et al., 2023). Genome‐mining bioinformatics tools like antiSMASH are very useful for discovering new natural products, such as antibiotics and anticancer compounds. In recent years, thanks to the latest advances in DNA sequencing, bioinformatics, and genome mining tools, a vast amount of data on natural product biosynthesis has been generated. This has encouraged artificial intelligence developers to focus on this type of data and develop machine‐learning tools for natural product discovery (Yuan et al., 2023). Recently implemented techniques, such as high‐precision single‐cell sequencing, microfluidics, and iCHIP diffusion chamber systems, have also greatly facilitated the analysis and screening of microorganisms for the production of new classes of antibiotics (Sherpa et al., 2015; Zhao et al., 2023). For example, the use of iCHIP diffusion chambers, which allow for in situ bacterial growth, has facilitated the discovery of two promising new antibiotics, lassomycin, and teixobactin. These have been shown to tackle difficult Gram‐positive bacterial infections (Qi et al., 2022; Zhu et al., 2019).

Finally, cell‐free protein synthesis systems have emerged as very promising platforms for target identification and drug discovery, complementing and advancing existing cell‐based expression approaches for natural product discovery (Tu et al., 2023). These systems allow the study of a wide range of biological reactions within a test tube, an approach that draws parallels to total synthesis from organic chemistry. However, since the reactions are biological, they do not require elevated temperatures, organic solvents, or heavy metal catalysts. The only requirements for these cell‐free systems are a cell extract, DNA, energy, and amino acids to catalyze coupled messenger RNA (mRNA) and protein synthesis in a one‐pot reaction (Moore et al., 2023). The importance of these systems is reflected in the fact that currently, multiple human clinical trials are in progress with cell‐free systems‐based products. In recent years, cell‐free protein synthesis technologies have grown from lab‐scale research tools to biopharmaceutical production at the “Good Manufacturing Practice” manufacturing scale (Zawada et al., 2022). In summary, regardless of the system used, natural sources will continue to play an important role in the identification of new antibiotics and other bioactive compounds in the future.

### 2.3 Other bioactive compounds produced by bacteria

#### 2.3.1 Antifungals

Unlike bacteria, fungi are eukaryotic organisms with a more complex cellular structure. They can exist in a unicellular form (yeast) capable of asexual replication or a filamentous form (mold) capable of both asexual and sexual replication. Fungal infections range from benign skin infections to life‐threatening conditions such as pneumonia, sepsis, and disfiguring diseases. While most fungi are effectively controlled by host immunity and can reside in a person for life, they can cause serious illness in some cases. Several antimicrobial compounds naturally produced by certain microorganisms can be utilized as antifungal drugs. In the past, the antifungal agents employed, both systemic and topical, were confined to amphotericin B and 5‐fluorocytosine, which were toxic and difficult to use. However, recent years have seen advancements in the treatment of fungal diseases through the availability of new bioactive agents and new formulations of older agents. These provide comparable, if not superior, efficacy to the previous ones, with significantly lower toxicity (Houšť et al., 2020). Amphotericin B (and its lipid formulations) exerts its antifungal action by either binding to ergosterol, the principal membrane sterol of fungi, or by directly damaging the fungal membrane. The binding of amphotericin B to ergosterol produces ion channels that destroy the osmotic integrity of the fungal cell membrane, leading to the loss of intracellular constituents and resulting in cell death. The binding of amphotericin B to cholesterol (a molecule very similar to ergosterol) accounts for most of the toxicity observed when amphotericin B is administered to humans, causing nephrotoxicity. Amphotericin B is effective against most fungi, including *Candida* and *Aspergillus* species (Baginski & Czub, 2009). The compound 5‐fluorocytosine (also known as flucytosine) has a different mechanism of action than amphotericin B and exerts its antifungal activity by interfering with the synthesis of nucleic acids (DNA and RNA) and proteins in the fungal cell (Murray et al., 2021). Contrary to amphotericin B, the antifungal spectrum of 5‐fluorocytosine is limited to some species of *Candida*, *Rhodotorula*, and some specific dematiaceous molds (Houšť et al., 2020). Worryingly, similar to the use of antibiotics, the widespread use of these compounds has generated resistance in many fungal species, including pathogenic *Candida* and *Aspergillus* strains (Thakur et al., 2024). In terms of the synthesis of these compounds, amphotericin B is of natural origin (produced by the bacterium *Streptomyces nodosus*) (Caffrey et al., 2001), while 5‐fluorocytosine is a synthetic compound synthesized in five steps starting from chloroacetamide (Ashe & Van Reken, 1977). Most of the antifungal drugs of natural origin currently in clinical use belong to either the polyene class, such as amphotericin B, nystatin, and natamycin, or to the echinocandin class, which includes caspofungin, anidulafungin, and micafungin (Baginski & Czub, 2009; Houšť et al., 2020). Echinocandins, which are not fully natural, are a highly selective class of semisynthetic lipopeptides that inhibit the synthesis of 1,3‐β‐glucans, important constituents of the fungal cell wall (Jospe‐Kaufman et al., 2024). Due to their mechanism of action, which is analogous to β‐lactam antibiotics in bacteria (i.e., inhibition of cell wall synthesis), this class of compounds is often termed the “penicillin of antifungals.” Several new compounds with antifungal activity, both natural and synthetic (e.g., nikkomycin Z, griseofulvin, olorofim, rezafungin, fosmanogepix, ibrexafungerp, opelconazole, and encochleated), have been developed through various scientific research programs and are in the process of being approved by major regulatory agencies, such as the FDA (Boutin & Luong, 2024). For instance, the synthetic compound olorofim (formerly known as F901318) was selected as one of the most efficient compounds against different species of the pathogenic fungus, *Sporothrix*, through iterative search and library screenings. However, this compound has not yet been approved by the FDA (Borba‐Santos et al., 2022). It is important to note that although a single microorganism can produce up to five structurally different compounds with antifungal activity in a single culture, the number of antifungal drugs approved for clinical use is not high due to the toxicity of most of these compounds (Houšť et al., 2020; Santos‐Beneit et al., 2022). Among the natural antifungal compounds currently under clinical evaluation, nikkomycin Z and griseofulvin are noteworthy. Nikkomycin Z is a uridine‐based secondary metabolite produced by *Streptomyces tendae* that inhibits the activity of fungal chitin synthase, essential for the formation of the fungal cell wall (Bormann et al., 1985). Griseofulvin, naturally produced by the soil fungus *Penicillium griseofulvum* (Oxford et al., 1939), inhibits fungal growth by interacting with microtubules within the fungal cell, resulting in the inhibition of mitosis. However, compared to these natural compounds, newer approved synthetic compounds, such as itraconazole and terbinafine, act more rapidly and provide greater efficacy (Murray et al., 2021).

#### 2.3.2 Antivirals

Hundreds of species of viruses can infect humans, leading to outcomes ranging from asymptomatic seroconversion to severe diseases, which can include respiratory failure, neurological damage, or hemorrhage (Kelley et al., 2023). Viral diseases can be as benign as the common cold or as deadly as Ebola. Unlike bacteria, viruses are obligate intracellular parasites that require the biosynthetic machinery and enzymes of host cells for replication. Therefore, inhibiting viral replication without also being toxic to the host is more challenging. Most antiviral drugs target viral‐encoded enzymes or virus structures that are crucial for replication. Other targets include those important for attachment, protein synthesis, assembly, penetration, and uncoating (Woolhouse et al., 2012). In addition, many native enzymes of the host that are necessary to produce the constitutive biomolecules of viral particles also constitute potential drug targets for tackling viral infections (Santos‐Beneit et al., 2021). Therefore, drugs targeting key human metabolic enzymes can be used to inhibit viral replication. For example, nucleoside and nucleotide analogs such as Tenofovir, Sofosbuvir or Ribavirin are often used as antiviral drugs (Nishijima et al., 2019). Although the search for antivirals began with the successful isolation of small molecule‐based compounds from microorganisms, such as certain antibiotics, almost all of the antiviral drug therapies currently in use are of chemical origin (Holmes et al., 1981; Velásquez et al., 2024). The screening of compounds of natural origin often resulted in lower antiviral activities in vivo than in vitro or a very high degree of toxicity for therapeutic applications (El Sayed, 2000). Current antiviral drugs are available for major viruses causing significant morbidity and mortality that provide suitable targets for drug action. However, unlike antibiotics, the activity of most antiviral drugs is limited to a specific virus. Antiviral drugs can treat a range of viruses, including Herpes simplex virus, Varicella‐zoster virus, Cytomegalovirus, Human immunodeficiency virus, Influenza A and B viruses, Respiratory syncytial virus, Hepatitis B and C viruses, Adenovirus, and Papillomavirus (Murray et al., 2021). With current advances in the fields of metagenomics and bioinformatics, it is expected that natural products (either from plant or microbial origin) will also play a central role in the discovery and development of new antiviral drugs in the near future (Aggarwal et al., 2023; Gabarin et al., 2023; Gabbianelli et al., 2023).

#### 2.3.3 Antiparasitics

Parasites are organisms that live on or inside a host and derive nutrients from that host. They exhibit substantial complexity and play a significant role in medicine. Although all parasites are eukaryotes, some are unicellular and others multicellular, and in some cases, they can also be considered microorganisms. Their size ranges from tiny protozoa of a few micrometers (i.e., just slightly larger than bacteria) to flatworms that can reach 10 m in length. Their life cycle is equally complex, with some establishing a permanent relationship with humans and others going through a series of developmental stages in various animal hosts (Murray et al., 2021). There are no treatments for all parasites, and the development of resistance to antiparasitic agents complicates the prevention and treatment of many infections involving these organisms (Ribeiro et al., 2023). The difficulties in treating parasitic diseases largely stem from the fact that both the cells of the human host and the parasite are eukaryotic and, therefore, share the same targets for a given compound. For this reason, most antiparasitic agents have to target pathways shared by both the parasite and the host. In most cases, antiparasitic activity is achieved by using compounds with differential susceptibility of functionally equivalent sites in the parasite and host or in the uptake or metabolic alteration of the drug. While traditional antiparasitic treatments based on the use of heavy metals are still in use, new agents have recently emerged that significantly improve the treatment of parasitic diseases. Examples of the chemotherapeutic differences between parasite and host that these new agents exploit include: (i) the inhibition of the folic acid pathway (some parasites are unable to use exogenous folate), exploited by pyrimethamine or trimethoprim‐sulfamethoxazole; (ii) interfering with neuromediators unique to parasites (i.e., exploited by diethylcarbamazine); (iii) accounting for drug‐concentrating mechanisms unique to the parasite (i.e., exploited by chloroquine); (iv) interacting with tubulin unique to parasites (i.e., exploited by benzimidazoles); (v) interfering with chloride channels (resulting in hyperpolarization of parasite cells, which causes death), exploited by the drug, ivermectin (Murray et al., 2021). In relation to antiparasitic drugs approved by the FDA (and other regulatory agencies) that are naturally produced by microorganisms, the sesquiterpenes (i.e., fumagillin, produced by Aspergillus fumigatus), avermectins (i.e., ivermectin, produced by *Streptomyces avermitilis*), and distinct inhibitors of protein synthesis (i.e., tetracycline and paromomycin, produced by Streptomyces spp.) are noteworthy. Sesquiterpenes, whose main member is artemisinin, are antimalarial compounds with the ability to significantly reduce parasite biomass. Artemisinin, unlike fumagillin (which inhibits RNA and DNA synthesis), reacts with the heme moiety, causing free‐radical damage to parasite membranes (Huang et al., 2023). Ivermectin, the main avermectin, blocks the neuromuscular action of the parasite and also inhibits the reproductive function of the adult female. Although ivermectin is widely used to control intestinal nematode infections in domestic and farm animals, its use in humans is primarily limited to the treatment of ocular and lymphatic filarial infections (Suvarna, 2023). Finally, the most important inhibitors of protein synthesis in bacteria that also exhibit antiparasitic activity are clindamycin, tetracycline, doxycycline, spiramycin, and paromomycin. Clindamycin and tetracycline are active against amebae and Babesia species. Doxycycline is used for the chemoprophylaxis of chloroquine‐resistant *Plasmodium falciparum* malaria. Spiramycin, as an alternative treatment to clindamycin, is used for the treatment of *Toxoplasma gondii* infections (toxoplasmosis). Finally, the aminoglycoside, paromomycin, is used as a secondary drug for treating amebiasis and giardiasis, and could be also useful for treating cryptosporidiosis (Murray et al., 2021).

#### 2.3.4 Immunosuppressants

Many of the immunosuppressive compounds currently available in the market are of natural origin and are fully synthesized by bacteria or fungi. These include cyclosporine, rapamycin, ascomycin, pimecrolimus, tacrolimus, and mycophenolate (Cen et al., 2024). For instance, cyclosporin is an 11‐amino acid cyclic nonribosomal peptide (undecapeptide) produced by the fungus *Tolypocladium inflatum* (Survase et al., 2011). On the other hand, tacrolimus, pimecrolimus, ascomycin, and rapamycin are macrolides produced primarily by the species *Streptomyces tsukubaensis* (Ordóñez‐Robles et al., 2017). These compounds belong to a broader group known as polyketides, whose biosynthesis shares chemical mechanisms and precursors with the biosynthesis of fatty acids (Hertweck, 2009). Tacrolimus (also known as FK506) is used in clinics to prevent graft rejection and to treat skin diseases (Ordóñez‐Robles et al., 2018). The use of tacrolimus in the treatment of patients with liver transplants has supplanted that of cyclosporine because tacrolimus is much more potent (up to 100 times more) than cyclosporine (Bellini et al., 2024; Haddad et al., 2006). Tacrolimus is one of the most effective immunosuppressants in the treatment against the rejection of transplanted organs (Bellini et al., 2024; Meier‐Kriesche et al., 2006). Unfortunately, the production yields of tacrolimus from the producing strains are very low, which significantly increases the cost of the final product (Kosec et al., 2012). Therefore, pharmaceutical companies have developed several strategies for culture media optimization, precursor feeding, and genetic engineering to increase the production of the compound. However, these have had limited success so far (Cen et al., 2024). This highlights the importance of microbial biotechnology and genetic engineering strategies for enhancing the production of valuable compounds.

#### 2.3.5 Vitamins

Among the most important vitamins for human consumption, riboflavin (vitamin B2) is primarily produced by two different microorganisms, *Eremothecium ashbyi* and *Ashbya gossypii*. To increase the yield of riboflavin, new and improved production processes have been developed using recombinant expression yeast models, such as *Candida albicans* (Sengupta et al., 2012). On the other hand, vitamin B12 is produced exclusively by bacteria and archaea, but not by fungi, plants, or animals. B vitamins, including vitamin B12, are crucial for protein metabolism in humans and, therefore, constitute a nutritional requirement for human health. Vitamin B12 aids in the formation of red blood cells and the maintenance of the nervous system. Thus, bacteria that synthesize vitamin B12 are very important and valuable sources for pharmaceutical companies. On an industrial scale, bacteria such as *Propionibacterium shermanii* or *Paracoccus denitrificans* are typically used for vitamin B12 production. The early stage of the *P. shermanii* fermentation is conducted under anaerobic conditions in the absence of the precursor 5, 6‐dimethylbenzimidazole. These conditions prevent vitamin B12 synthesis and allow for the accumulation of the intermediate, cobinamide. The culture is then aerated, and dimethylbenzimidazole is added to convert cobinamide to vitamin B12. Alternative industrial procedures are also possible. For example, when using *P. denitrificans* fermentation, the entire process is carried out under low oxygen concentrations (Gardner & Champagne, 2005; Kryl et al., 2023).

#### 2.3.6 Anticancer drugs

Cancer is one of the major deadly diseases worldwide. Various natural compounds synthesized by plants and microorganisms are used to combat cancer through different mechanisms. More than 60% of the antineoplastic drugs approved by the FDA come from natural sources (Newman & Cragg, 2020). These natural compounds belong to different chemical classes, including terpenoids, alkaloids, flavonoids, and other polyphenols, among others (Asma et al., 2022). Many excellent reviews describe the different types of anticancer drugs (natural, semisynthetic, or synthetic) that are used (or in development) for the treatment of cancer (Giraud et al., 2023; Kroemer et al., 2023; Ma & Adjei, 2009; Meltzer & Helman, 2021; Moreau Bachelard et al., 2021). In this section, a summary of the main classes of natural compounds used for cancer treatment is provided, with a special focus on those natural products synthesized by bacteria. Alkaloids are important plant secondary metabolites that produce many health benefits and treat many diseases, including cancer (Qin, You, et al., 2022). Some alkaloids include vinblastine, vinorelbine, vincristine, vindesine, vinflunine, veratridine, and berbamine (Asma et al., 2022). These compounds are used to treat several types of cancer and can inhibit different cancer pathogenesis pathways (Efferth & Oesch, 2021). Many works in the literature have reported that alkaloids can regulate cell death by targeting the classical apoptosis and autophagic cell death signaling pathways, as well as the crucial signaling pathways of other regulated cell death subroutines, such as ferroptosis, mitotic catastrophe, necroptosis, and anoikis (Qin, You, et al., 2022; Song et al., 2021). Terpenoids, similar to alkaloids, are a large group of natural compounds with broad anticancer properties that include different categories (i.e., mono, di, tri, tetra, and sesquiterpenoids) (Chopra et al., 2021). On the other hand, flavonoids (the major group of polyphenols present in plants with medical value), categorized as flavanols, flavones, flavanones, isoflavones, chalcones, and anthocyanidins, have also been shown to display important anticancer activity (Malla et al., 2022). In addition to flavonoids, other natural groups of polyphenols with anticancer properties are stilbenes, curcuminoids, lignans, and phenolic acids (highlighting the compounds resveratrol, curcumin, magnolol, and arctigenin) (Montané et al., 2020). Among the bacterial strains producing anticancer compounds, *Streptomyces* species stand as the most proficient producers of anticancer drugs (Bahrami et al., 2022; Law et al., 2020; Watve et al., 2001). Examples of natural anticancer compounds produced by bacteria include bleomycin (a mix of glycopeptides produced by *Streptomyces verticillus*) (Umezawa et al., 1966), dactinomycin (a nonribosomal peptide produced by *Streptomyces chrysomallus*) (Shafer et al., 1982), mitomycin C (a quinone produced by *Streptomyces caespitosus*) (Tomasz, 1995), and doxorubicin (an anthracycline produced by *Streptomyces peucetius*) (Lomovskaya et al., 1999). Furthermore, these natural microbial compounds have been modified and developed into important drug leads such as doxorubicin (Doxil), daunorubicin (DaunoXome), dactinomycin (Cosmegen), mitomycin C (Mutamycin), bleomycin (Blenoxane), pingyangmycin (Bleomycin A5), streptozotocin (Zanosar), and the semisynthetic derivatives of the doxorubicin and daunorubicin compounds, valrubicin (Valstar) and idarubicin (Idamycin), respectively (Bahrami et al., 2022; Girigoswami et al., 2023; Law et al., 2020; Taliento et al., 2023). Streptomycetes are also the original producers of other anticancer drugs in development, such as isomigrastatin and dorrigocin (Lo Re et al., 2015). Hence, the pharmaceutical and medical significance of Streptomycetes is immeasurable and unparalleled among all other types of microorganisms.

### 2.4 Most important therapeutic compounds produced by yeast and algae

#### 2.4.1 Biotherapeutic products from yeast

Yeasts are unicellular, ubiquitous eukaryotic organisms traditionally isolated from soil, water, plants, honey, and food stocks (Pang et al., 2022). Numerous yeast and yeast‐derived products are produced and marketed as food supplements, functional foods, and pharmaceuticals, including anticancer and antimicrobial compounds (Roohvand et al., 2017). For instance, farnesol, a molecule present in essential oils and also produced by *Candida albicans* as a quorum‐sensing component, displays inhibitory properties in the formation of microbial biofilms and synergism with antimicrobials used in clinical practice (Costa et al., 2021). The presence of commensal yeast species in the human gut suggests that these organisms have the potential to benefit the host. Species of *Saccharomyces cerevisiae* and *Saccharomyces boulardii* are often used as probiotics (Sen & Mansell, 2020). Moreover, several studies using animal hosts (and in clinical trials) suggest that *S. boulardii* can be used as a biotherapeutic in humans, especially to alleviate symptoms from gastrointestinal tract infections (Everard et al., 2014; Koon et al., 2016; Sen & Mansell, 2020). One of the most significant applications of these Generally Recognized As Safe (GRAS) yeasts is the production of therapeutic proteins using their cells, particularly those of *Pichia pastoris* and *S. cerevisiae* species, as “cell factories.” Yeast cells are also employed for the production of subunit vaccines, and due to the immunomodulatory properties of their β‐glucans cell wall components, whole yeast cells are also in development as new “live vaccine” platforms. Indeed, the ability of yeast cell wall β‐glucans to stimulate the immune system, combined with the possibility of using these organisms as heterologous expression platforms (for expressing pathogen or tumor antigens), has expanded their application as promising novel biotherapeutics, termed “whole yeast vaccines” (Roohvand et al., 2017). Furthermore, yeasts offer useful characteristics as eukaryotic model organisms due to their ease of growth and their wide range of possibilities for genetic manipulation. For example, “yeast humanization,” ranging from a single point mutation to substitution of a gene (or even a complete pathway) by human counterparts, has greatly expanded promising yeast biomedical applications, including screening of effective drugs and studies on human diseases, such as cancer. Humanized yeasts combine the classical advantageous features of a “microbial eukaryote” with advanced human cellular processes, allowing the production of functional and stable therapeutics at lower prices compared to mammalian (Chinese hamster ovary [CHO]) production‐based systems (Roohvand et al., 2020).

#### 2.4.2 Biotherapeutic products from algae

Algae are eukaryotic aquatic organisms that offer a wealth of beneficial products for human nutrition and health. They are rich in omega‐3 fatty acids (i.e., eicosapentaenoic acid and docosahexaenoic acid), essential amino acids, antioxidants (i.e., carotenoids and flavonoids), vitamins (i.e., vitamins A, C, E, and K), and minerals. These nutrients are important for proper heart and brain functions, reducing the risk of chronic diseases, and supporting overall well‐being. In particular, seaweed, a diverse group of marine algae, is well recognized not only for its rich nutritional composition but also for its ability to produce various bioactive compounds. As such, it is considered a nutraceutical ingredient (Cotas et al., 2024). One of the most important nutraceuticals produced by algae is astaxanthin. This red‐colored keto‐carotenoid compound has excellent antioxidant properties and has emerged as a promising therapeutic drug. Astaxanthin has been shown to have a positive effect against various significant diseases and disorders such as obesity, diabetes, neurodegenerative syndromes, and cancer, among others (Patil et al., 2022). Indeed, several species of algae are important sources of different compounds (i.e., fucoxanthins, phlorotannins, phytosterols, and fucoidans) with demonstrated anticancer activity against a wide range of cancers (i.e., pancreatic, lung, breast, cervical, colorectal, liver, gastric, leukemic, and melanoma) (Nova et al., 2023). Moreover, various natural products from algae (such as cyanovirin, scytovirin, and microvirin) have been shown to display antibacterial and antifungal properties. Other algae‐derived compounds, such as lectins and sulfated polysaccharides, have also been reported to have antiviral activity (Afzal et al., 2023).

## 3 PROTEINS AND PEPTIDES AS THERAPEUTIC OPTIONS

In recent decades, protein‐based therapeutic agents have become highly successful in clinics, leading to new paradigms in disease treatment. Recombinant antibodies are among the most proficient examples of these agents. Proteins have emerged as competitive alternatives to historically used small molecule‐based medicines (Randall & Davies, 2021).

### 3.1 Genuine unmodified bacterial nonrecombinant proteins

Bacteria not only produce secondary metabolites (small molecules) that are used as therapeutic drugs, such as antibiotics (as described in Section 2.2), but also numerous extracellular enzymes. These include chitinases, lipases, amylases, proteases, and cellulases, which are very useful for the industry and other technological fields (Challis & Hopwood, 2003). However, only a few genuine unmodified bacterial proteins are used directly as therapeutic drugs in the clinic. Some of the few exceptions are streptokinase, collagenase, and l‐asparaginase. Streptokinase is naturally produced by *Streptococcus* spp. bacteria, which use this enzyme to break up blood clots, allowing them to spread from the initial site of infection (Wang et al., 1995). In the clinic, streptokinase (trade name Streptase) is used to treat acute evolving transmural myocardial infarction, pulmonary embolism, deep vein thrombosis, arterial thrombosis, and occlusion of the arteriovenous cannula by converting plasminogen to plasmin (Leader et al., 2008). Collagenase is obtained from *Clostridium histolyticum* cultures. This enzyme allows the bacterium to digest collagen in the necrotic base of wounds (Rao et al., 1975). In the clinic, collagenase (trade name Santyl) is used to treat chronic dermal ulcers (Leader et al., 2008). Finally, l‐asparaginase is naturally produced by *Escherichia coli*, which displays asparaginase activity, allowing the removal of available asparagine from serum (Clavell et al., 1986). In the clinic, l‐asparaginase (trade name ELSPAR) is widely used to treat acute lymphoblastic (or lymphocytic) leukemia (ALL) and lymphoblastic lymphoma (LBL) (Leader et al., 2008; Sato et al., 2023; Siegel et al., 2018; Teachey & Pui, 2019). However, in general, with few exceptions, most of the genuine unmodified therapeutic proteins for clinical use are harvested from plasma, human tissues, or other eukaryotic cells, rather than from bacteria (Leader et al., 2008).

### 3.2 Recombinant proteins

In 1922, the first therapeutic protein other than antibodies, namely insulin, was purified from animal pancreas and administered to patients with diabetes mellitus. However, the availability, cost, immunogenicity, and risk of diseases being transmitted from the producing tissue limited the use of animal‐derived insulin (Mathieu et al., 2021). A breakthrough occurred in 1982 when recombinant DNA technology was used to produce Humulin (human insulin) in the bacterial host *E. coli* (Goeddel et al., 1979). The successful use of recombinant DNA technology helped to overcome challenges with both scale‐up and immunogenicity of animal‐derived proteins. After Humulin (the first FDA‐approved, recombinant, protein‐based therapeutic), most protein therapeutics approved for clinical use were also recombinant. Recombinant proteins used in the clinic include, among others, recombinant interleukins, interferons, hormones, growth factors, blood clotting factors, thrombolytic drugs, and many different types of enzymes for treating a wide range of diseases (Miao et al., 2024). Engineering recombinant proteins and their heterologous expression in bacterial models not only provide a ready source of the products but also enable modifications to be made in their structure that can maximize clinical potential. For example, additional glycosylation sites can be added to the protein or specific protein domains can be fused. With the bacterial host, *E. coli*, the produced protein is not glycosylated. Therefore, if glycosylation is important, this bacterium cannot be used, and other expression models, such as *S. cerevisiae* or *P. pastoris* systems, should be utilized (Lakowitz et al., 2018). In recent decades, genetic engineering has led to the development of new recombinant therapeutic proteins with optimized pharmacokinetics and hundreds of them are in clinical trials for the therapy of cancers, immune disorders, infections, and differential diagnosis (Shah et al., 2023).

### 3.3 Antibodies

Antibodies are a series of immunoglobulin molecules produced by B‐lymphocytes as part of the adaptive immune response when encountering a foreign molecule. These antibodies react against a specific antigen, each identifying a different epitope on an antigen. The most commonly used antibody isotype is the immunoglobulin IgG, which can be either polyclonal or monoclonal. Polyclonal antibodies contain a heterogeneous mixture of IgGs (i.e., synthesized from different immune cells) against the whole antigen (i.e., having an affinity for the same antigen but against different epitopes). In contrast, monoclonal antibodies are composed of a single IgG against one epitope (i.e., made using identical immune cells) (Mitra & Tomar, 2021). Monoclonal antibodies allow for higher specificity to a single epitope, which is also reflected in low cross‐reactivity (Dos Passos et al., 2023). Although polyclonal antibodies were a component of the first successful immunosuppressive regimens in the 1960s, for clinical applications, monoclonal antibodies are a better solution. This is because, among other advantages, they show less chance of cross‐reactivity due to the recognition of multiple epitopes (as polyclonal antibodies do) (Henrique et al., 2022). For general research applications, however, the advantages of polyclonal antibodies typically outweigh the few advantages that monoclonal antibodies provide. Polyclonal antibody production is inexpensive and relatively quick to produce, uniquely involving the repeated immunization of an animal with the desired antigen and the bleeding of the animal when a sufficient concentration of the antibody is obtained (Mitra & Tomar, 2021). On the other hand, the production of monoclonal antibodies requires hybridoma cell lines, a technique that was introduced by Köhler and Milstein in 1975. In this process, antibody‐producing B‐lymphocytes are fused with immortal cancerous cell lines such as myeloma cells, creating an immortal hybrid cell line that produces antibodies limitlessly (Köhler & Milstein, 1975). The increasing importance of monoclonal antibodies is apparent as these proteins have become the predominant treatment modality for various diseases over the last decades. In fact, by 2023, there were nearly 1200 monoclonal antibody therapeutics in clinical studies and around 175 in regulatory review or approval (Kaplon et al., 2023). This points to a shift toward precise and personalized medicine using these types of therapeutics (Dos Passos et al., 2023). The next generation of monoclonal antibodies has been represented by recombinant antibodies, which are very promising alternatives to the classical ones as they allow for multiple engineering possibilities that can be performed to alter and improve the properties of the monoclonal antibody. Recombinant antibodies are generated in vitro using synthetic genes that are usually expressed from a plasmid or a sequence integrated into a stable cell line. The synthetic genes cloned in the producing cells encode the heavy and light chains of the antibody. When translated into protein, these chains are assembled into a fully functional antibody that can be used in the same way as antibodies made from animals or hybridomas (Basu et al., 2019). Therefore, recombinant antibodies are produced without immunizing any animals or cultivating any hybridomas. Recombinant antibodies can be cloned from any species of antibody‐producing animals, with the only requirement being knowledge of the sequence of the genes for antibody expression. After the antibody of interest has been cloned into an expression plasmid, the plasmid can be introduced into host cells, such as bacterial, yeast, or mammalian cells, for antibody production and subsequent purification. To determine the sequence to be cloned, mass spectrometry can be used to identify the amino acid sequences of a given antibody. With this information, the synthetic genes that code for those amino acids can be designed (Tran et al., 2016). On the other hand, if a hybridoma cell line is used, the antibody can be made recombinant by sequencing the DNA of the hybridoma cell line and subsequently cloning a gene encoding the identified sequence (Andrews et al., 2019). Finally, another method to produce recombinant antibodies is by selecting antigen binding from recombinant antibody libraries (Wang et al., 2023). Chimeric and humanized antibodies are two types of nonhuman recombinant antibodies whose sequences have been obtained from the nonhuman immune system (such as from mice). The key difference between a chimeric and a humanized antibody is that a chimeric antibody is made up of domains of different species and carries a larger stretch of nonhuman protein, while a humanized antibody is an antibody that has been modified to increase its similarity to antibody variants produced naturally in humans (Mihaylova et al., 2024). The International Nonproprietary Names (INN) for humanized antibodies end in “zumab,” as in Bevacizumab, Natalizumab, or Trastuzumab. Treatments with recombinant antibodies have revolutionized medicine and led to new paradigms in disease treatment. However, while efforts to identify antibodies with direct antibacterial activity have been challenging, other antibody‐based approaches have shown promise in tackling infectious diseases. For example, MedImmune has developed a bispecific antibody that targets the virulence factor PcrV and the exopolysaccharide Psl of *Pseudomonas aeruginosa*. This antibody is currently in clinical development to tackle difficult *P. aeruginosa* infections (Ali et al., 2019). In addition, other developments blend the advantages of using an antibody and a small molecule, such as an antibiotic. For instance, Genentech has developed a novel antibody‐antibiotic conjugate to combat *Staphylococcus aureus* infections. In this strategy, the antibody binds to the bacterium, but the antibiotic is only activated once it penetrates the host cells that have absorbed the bacterium due to the specific action of the antibody (Lehar et al., 2015; Peck et al., 2019). Blended approaches have also been developed to combat cancer. For example, the biotechnological company Sesen Bio (now Carisma Therapeutics) developed a targeted fusion protein that binds a monoclonal antibody and a bacterial toxin. The developed product, Vicineum (also known as VB4‐845), was successfully tested for treating Bacillus Calmette‐Guérin (BCG) unresponsive nonmuscle invasive bladder cancer, although the compound has not yet been approved by the FDA (Dickstein et al., 2018). The recombinant VB4‐845 fusion protein (Vicineum) was made up of a monoclonal antibody (anti‐EpCAM) linked to a truncated form of *Pseudomonas aeruginosa* exotoxin A (ETA[252–608]KDEL), which has previously been shown to inhibit protein synthesis and reduce the viability of Ep‐CAM‐positive carcinoma cells of diverse histological origins (Di Paolo et al., 2003). Vicineum is just one example, as many similar immunotoxins are also in development for treating other types of cancer or diseases. One of the challenges of these protein‐based therapies is that they might not be suitable for brain drug development programs because the crossing of the blood‐brain barrier is quite restricted for large molecules such as proteins and antibodies (Bruell et al., 2005; Martin‐Killias et al., 2011).

### 3.4 Toxins

Many bacterial pathogens produce toxins that damage host cells through various mechanisms, including creating holes in cell membranes or damaging DNA. However, in some cases, toxins can be employed to promote human health or to treat several types of cancer. Many toxins are used as therapeutic agents either directly or through the development of other therapeutic agents, such as the immunotoxins described in Section 3.3. One of the most therapeutically used toxins is produced by the bacterial species, *Clostridium botulinum*. Botulinum toxin type A (trade name, Botox) is a nonrecombinant therapeutic protein of natural origin obtained from *C. botulinum*. It provides a novel function when applied to humans, distinct from its native activity within the bacterial cell. In human cells, the enzyme cleaves SNAP‐25 (a critical protein for the fusion of plasma membrane and synaptic vesicle) at neuromuscular junctions to disrupt the SNARE complex (the motors that drive the biological fusion of two membranes) and prevent acetylcholine release, causing flaccid paralysis (Blasi et al., 1993; Oates et al., 1991). Botox is used in the clinic to treat many types of dystonia, particularly cervical, and also for cosmetic uses (Wheeler, 1997). Botulinum toxin type B is another toxin produced by *C. botulinum* (sharing a similar function to the tetanus toxin produced by Clostridium tetani). Instead of cleaving the SNAP‐25 protein, it specifically cleaves synaptobrevin (another membrane protein of synaptic vesicles) (Schiavo et al., 1992). Similar to botulinum toxin type A, toxin type B disrupts the SNARE complex and prevents acetylcholine release, causing flaccid paralysis (Blasi et al., 1993; Oates et al., 1991). Botulinum toxin type B (trade name, Myoblock) is employed for almost the same uses as Botox (Wheeler, 1997). Other promising therapeutic options based on toxins are those that leverage the existence of toxin‐antitoxin (TA) systems in pathogenic bacteria. These systems can be used as new targets to combat bacterial infections (i.e., uncontrolled toxin expression elicits a bactericidal effect). TA systems, with type II TA systems being the most well‐characterized, are present in many pathogenic bacteria, including *Mycobacterium tuberculosis*, *Staphylococcus aureus*, and *Neisseria gonorrhoeae*. The TA systems usually consist of two genes that encode a toxic protein (targeting an essential cellular process) and an antitoxin that counteracts the activity of the toxin. When functioning correctly, intracellular toxins bind to antitoxins, forming a protein complex that protects the bacteria from damage. However, if the antitoxin is degraded by proteases, the toxin is released and the bacterium is harmed. Prevention of the formation of TA complexes (e.g., by degrading the antitoxin) can occur naturally (under specific stressful conditions) or artificially by using different strategies that disrupt or prevent the formation of the TA complex itself (including protein or RNA‐based approaches) (Równicki et al., 2020). Therefore, bacterial TA systems stand out as promising new antimicrobial targets in pathogenic bacteria that can be tackled using appropriate genetic or chemical tools.

### 3.5 Antimicrobial peptides (AMPs)

AMPs are emerging as an alternative to traditional antibiotics for treating drug‐resistant bacteria (Mba & Nweze, 2022). It's important to distinguish between AMPs and nonribosomal antimicrobial peptides (NRAMPs). AMPs are proteins synthesized from genes through the standard process of protein translation by ribosomes. In contrast, NRAMPs are synthesized via multifunctional enzyme systems, such as nonribosomal peptide synthetases (NRPs) and polyketide synthases (PKs) (Ji et al., 2024). NRAMPs are small molecules (typically <3 kDa) that include lipopeptides, glycopeptides, and polypeptides, which have been described in Section 2.2. On the other hand, AMPs are larger molecules composed of different amino acids (typically between 10 and 60; average 33) (Huan et al., 2020). AMPs are attracting substantial attention due to their safety and broad spectrum of antimicrobial activity, with slower development of antimicrobial resistance due to their unique mode of action and multitarget effects. However, the high production cost and low biological activity of these compounds have slowed their development in the last decade (Xuan et al., 2023). Currently, with the advancement of computer technology and artificial intelligence, a new direction in the study and application of AMPs has emerged. Databases of thousands of natural and synthetic AMPs are now available, facilitating computationally aided de novo generation and optimization of these compounds. Recent studies have shown the design principles that can be applied to identify stable, active, and nontoxic AMPs for treating infections, as well as to further increase the efficiency of existing compounds (Wang, 2023). Most of the AMPs developed to date are natural compounds derived from animals, plants, and microorganisms (Ji et al., 2024). Microbial‐derived AMPs include, among others, nisin (from *Streptococcus lactis*), ε‐polylysine (from *Streptomyces albulus*), and pediocin PA‐1 (from *Pediococcus pentosaceus*). Nisin is one of the major representatives of the compounds known as “lantibiotics” (a class of polycyclic peptide antibiotics that are ribosomally synthesized and posttranslationally modified) (Kaletta & Entian, 1989). The term “lantibiotic” was introduced in 1988 as an abbreviation for lanthionine‐containing antibiotic peptides. Lanthionine is composed of two alanine residues that are crosslinked on their β‐carbon atoms by a thioether linkage (Schnell et al., 1988). Nisin binds to lipid II (a cell wall precursor lipid component of target bacteria) and disrupts cell wall formation, killing the bacterial cell (Kaletta & Entian, 1989). However, despite nisin being approved for food preservation, it is not yet used in the clinic as an antimicrobial drug (Christmann et al., 2023).

## 4 VACCINES

Depending on the pathogen (i.e., bacteria or virus) being targeted, and several other factors to consider, distinct vaccine technologies are used to generate an effective immune system response. There are different vaccine technology platforms, each with its benefits, including the traditional ones (see below) and the newer vaccine platforms based on messenger RNA, genetic modified viruses or recombinant peptides and nanoparticle conjugates. This section focuses specifically on the platforms in which microbial biotechnology plays a significant role, such as toxoids, subunits, and live attenuated vaccines. The most effective immune responses are generally produced in response to antigens present in a live organism. Two different classes of vaccines use the whole germ (virus or bacterium). The “inactivated vaccines” use a killed version of the germ, and the “live‐attenuated vaccines” use an attenuated or weakened version of the germ. Both types of vaccines are relatively inexpensive to make and produce a robust enough immune response (Poria et al., 2024). However, an antigen does not necessarily have to be present in a whole organism to produce an immune response. In this sense, “subunit vaccines” are made from a fragment of a pathogen rather than from the whole organism. Depending on the nature of the fragment, different types of vaccines can be made. When a purified protein, or even a single epitope of a protein, is used, the technology is known as “protein‐based vaccine” (Poria et al., 2024). On the other hand, when the component administered is a polysaccharide, the technology is known as “polysaccharide vaccine” and, if the polysaccharide component is stuck to a protein, the technology receives the name of “conjugate vaccine” (Poria et al., 2024). To tackle bacterial infections, different vaccines based on distinct technologies have been developed (see Table A2). Among these vaccines, the Bacillus Calmette‐Guérin (BCG) live‐attenuated vaccine (generated via serial subculturing of *Mycobacterium bovis*) provides a versatile therapeutic option for treating both tuberculosis and Non‐Muscle‐Invasive Bladder Cancer (NMIBC) (Basak et al., 2021; Herr, 2012). Although new vaccines (different from BCG) have been developed against *Mycobacterium tuberculosis*, BCG vaccine is still the gold standard treatment for high‐grade and high‐risk NMIBC (kaufmann, 2023; Zhou & Zhang, 2023). However, some bladder cancer cells have become unsusceptible to this treatment, and therefore, new chemotherapeutic alternatives are required (Lopez‐Beltran et al., 2024). Finally, there are new vaccines recently approved or in development against different important pathogens, such as *Shigella* spp. and enterotoxigenic *E. coli* (ETEC) strains (Hui Xian et al., 2022; Mirhoseini et al., 2018). Examples of distinct types of bacterial vaccines are given below.

### 4.1 Toxoid vaccines

Certain bacterial diseases are caused by toxins synthesized by the bacterium, rather than the bacterium itself. This is the case with infections caused by bacterial pathogens such as *C. tetani* (which causes tetanus) and *C. diphtheriae* (which causes diphtheria) (Murray et al., 2021). Immunizations for these types of pathogens can be achieved using vaccines containing inactivated versions of the bacterial toxins, known as toxoids. When immune cells encounter these toxoids, they generate antibodies that can recognize and neutralize the actual toxins during infection. Therefore, toxoid vaccines use inactivated toxins to target the toxic activity created by the bacteria, rather than targeting the bacteria itself. Toxoids can also be used to make conjugate vaccines (Espinosa‐Viñals et al., 2017). For example, to protect against *Streptococcus pneumoniae*, a polysaccharide is conjugated to the diphtheria toxoid and administered, resulting in a significant immune reaction against seven serotypes of the pathogen (Curry et al., 2023).

### 4.2 Bactofection

A large number of nucleic acid‐based therapies (RNA or DNA) are currently in clinical trials and are also being approved. However, delivering these molecules to the target site is challenging and relies on the use of appropriate delivery systems. Several delivery systems have been developed and investigated to overcome some of these challenges. For example, invasive bacteria such as *Salmonella, Listeria, Yersinia, Shigella*, and *Mycobacterium* have been used as vaccine vectors, proving capable of generating powerful humoral and cellular immune responses (Li et al., 2020). There are several advantages to using recombinant bacterial vector systems for vaccine development. First, most bacterial systems can easily incorporate large target sequences via plasmid or cosmid extrachromosomal DNA components, which constitutes inexpensive procedures. Second, live bacteria have innate adjuvant properties, which enable both the delivered molecules and the bacteria themselves to stimulate humoral and cell‐mediated immune responses (Lee & Kim, 2022). In this direction, “bactofection,” a term that defines the use of bacterial vectors to deliver foreign genes into host cells, constitutes a promising vaccine strategy for allowing the introduction of antigens and other molecules, such as cytokines, into specific target cells. The therapeutic bacteria that have been most frequently engineered to be used as bacterial delivery systems, and already used in human clinical trials, are *Salmonella enterica* and *Listeria monocytogenes* (Howell & Forbes, 2022; Song et al., 2023). Other bacterial vaccines in development are those using *Mycobacterium paragordonae* and *Lactiplantibacillus plantarum* (Lee & Kim, 2022). Genetic engineering of these bacterial systems offers a new landscape for developing novel vaccines, such as those known as “oral replicon‐based RNA vaccines.” These types of vaccines have been made possible by the exploitation of a bacterial vaccine (such as an attenuated *Salmonella* mutant strain that does not cause disease) and an alphaviral replicon (which provides a self‐amplifying RNA that is expected to improve the efficacy of mRNA vaccines) (Jawalagatti et al., 2022; Jawale et al., 2012). The bacterial nature of these vaccines allows for their nasal or oral application. Indeed, the nasal or oral use of bacterial vaccines for immunizing animal models against viral infections (such as avian influenza) has been widely approached by different research groups. For example, an attenuated *Salmonella*‐based vaccine (delivered either intramuscularly, nasally, or orally) was shown to protect chickens against heterologous pathogenic avian influenza viruses and to elicit efficient humoral and cell‐mediated immune responses, independently of the route of vaccination (Hyoung et al., 2017; Kim et al., 2018). The nature of *Salmonella* cells presents several advantages when the bacterium is used either as an oral or nasal vaccine due to its ability to invade and proliferate into antigen‐presenting cells (i.e., dendritic cells and macrophages) and to elicit potent systemic and mucosal immune responses against digestive and respiratory pathogens (Jawalagatti et al., 2022). This type of oral and nasal bacterial vaccine is also in development for preventing SARS‐CoV‐2 infection. In a recent study, oral administration of a recombinant attenuated *Salmonella* strain (expressing the full‐length spike gene of SARS‐CoV‐2) was shown to exert a protective effect against SARS‐CoV‐2 infection in a rat model, suggesting the feasibility of a COVID‐19 oral bacterial vaccine also for humans (Zhu et al., 2022). The existence of other licensed oral attenuated bacterial vaccines, such as the licensed *Vibrio cholerae* vaccine known as Vaxchora (Hui Xian et al., 2022; McCarty et al., 2022), increases the possibility of adopting such vaccines in the clinic in the near future. However, more studies using *Salmonella* or other relevant bacterial species in suitable clinical models are necessary to validate this technology.

## 5 MICROBIOTA, PROBIOTICS, AND FECAL TRANSPLANTS

Until birth, the human fetus resides in a highly protected and virtually sterile environment. However, this situation rapidly changes when the infant is exposed to bacteria, archaea, fungi, and viruses from the mother, other close contacts, and the environment. In the ensuing years, communities of organisms (i.e., microbiota) will form on the surface of the skin, the nasal passages, the oral cavity, the intestines, and the genitourinary system, which will vary with age and other factors (McCallum & Tropini, 2023). The term “microbiota” describes the range of microorganisms found in a multicellular organism, including commensal, mutualistic, and pathogenic microorganisms. “Microbiome,” on the other hand, refers to the collection of genomes of all the microorganisms present in a specific niche, including the human body (Walker & Hoyles, 2023). In the near future, with faster and cheaper DNA sequencing techniques and improved computational approaches, analysis of a person's microbiome may become a routine test to predict and treat a wide variety of diseases.

### 5.1 Importance of human microbiota

There is growing evidence that the microbiota plays many functional roles in human health, including the occurrence of host diseases or the maintenance of good health (Han et al., 2024). Some infections can alter the normal composition of the microbiota. For example, the human immunodeficiency virus (HIV), in addition to its chronic effect on the immune system, induces a compositional shift in the gut microbiota. This change in composition includes the enrichment of certain types of bacterial populations that are either pro‐inflammatory or potentially pathogenic, and their abundance correlates with the immune status (Mutlu et al., 2014). This raises the question of whether, conversely, the gut microbiota can contribute to disease progression. What is known today is that indeed, many disorders are linked to changes in the composition of the gut microbiota (Han et al., 2024). For example, inflammatory bowel disease, a chronic inflammatory disorder of the gastrointestinal tract, has been associated with dysbiosis in the composition and function of the gut microbiota, among other examples (Luo et al., 2022).

### 5.2 Oral probiotic therapy

Probiotics, understood as beneficial live microorganisms, have emerged as an effective strategy for regulating gut microbiota disorders. The increased awareness of the impact of dietary patterns on human health has led to the emergence of terms such as “probiotics,” “prebiotics,” and “symbiotics” (probiotics + prebiotics). Probiotics are a mixture of live microorganisms (e.g., *Lactobacillus* and *Bifidobacterium bacteria*) contained in foods (i.e., yogurt, kefir) or supplements (in capsules for ingestion) that produce beneficial properties for health. Prebiotics, on the other hand, are foods (e.g., high‐fiber food) that act as beneficial nutrients for human microbiota. When ingested, probiotics colonize and proliferate in the intestine, albeit temporarily. Probiotics have been used to treat diarrhea associated with *Clostridium difficile* and to provide protection against *Salmonella* and *Helicobacter pylori* infections, as well as a treatment for reducing dental caries, childhood atopic dermatitis, and autoimmune diseases (Rangel‐Torres et al., 2022). However, clear evidence of the effectiveness of probiotics in treating many of these diseases has not yet been demonstrated. Nevertheless, because bacteria can be engineered as living therapies that sense and respond to environments (which can also colonize niches in the gastrointestinal tract, mouth, lung, skin, or tumors), modulation of the microbiota with these living agents should be considered a promising approach for treating diseases. Researchers are developing carefully designed “smart probiotics” that might serve as important adjuvants to medical treatment in the coming years (i.e., oral probiotic therapy). Among these strategies, encapsulation technologies have emerged as a promising solution to address the challenges of delivering probiotics to the digestive tract (Harimoto et al., 2022). Two main encapsulation strategies for probiotics are in development: “microencapsulation” and “single‐cell modification.” Microencapsulation systems have been constructed using various methods such as drying, extrusion, complex coacervation, and emulsification (Ahmad et al., 2021). Single‐cell modification has been developed based on physical, chemical, and biological methods, creating diverse types of nanocoatings, including lipopolysaccharides, capsular polysaccharides, lipid membranes, proteins, and modified polymers (i.e., matching the efficacy of conventional microcapsules) (Han et al., 2024). The advantage of the single‐cell modification method is that it not only protects probiotic bacteria from environmental factors without compromising their activity and function but also provides additional functions to the bacteria, such as strong adhesion, antioxidant activity, and immunomodulatory capabilities (Yang, et al., 2022).

### 5.3 The use of live‐therapeutic bacteria for cancer treatment

**Figure 3:**
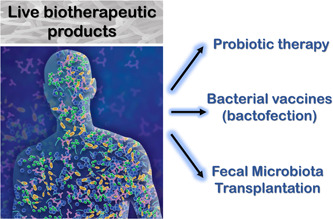
Live therapeutic products. Biotherapeutics, a new class of therapeutics, utilize live bacteria to prevent and combat various human diseases. Probiotic bacteria can be engineered to deliver therapeutic compounds into human cells. Conversely, invasive bacteria such as Salmonella, Listeria, Yersinia, Shigella, and Mycobacterium have been employed as vaccine vectors, demonstrating their ability to generate potent humoral and cellular immune responses (a process known as bactofection). Fecal Microbiota Transplantation has already received approval from various regulatory agencies, including the FDA, for the treatment of recurrent Clostridium difficile infections.

Although various types of cancer, mainly gastric carcinomas, are caused by bacteria (such as *Helicobacter pylori*, *Campylobacter jejuni*, or *Porphyromonas gingivalis*), several studies have shown that bacteria are also capable of treating cancer due to their ability to produce and release highly toxic chemicals with anticancer properties. Live attenuated strains of *Salmonella* are the most commonly used bacterial species in clinical trials for cancer treatment, among other reasons, because *Salmonella* cells can invade hypoxic tumor sites, which is crucial for the destruction of tumor cells. Other Gram‐negative bacteria belonging to the genera *Pseudomonas, Klebsiella, Caulobacter, Escherichia*, and *Proteus* have also been frequently employed to destroy tumors through different mechanisms (Mills et al., 2022). Although Gram‐positive bacteria, such as lactic acid bacteria (i.e., *Bifidobacterium*, *Listeria*, *Streptococcus*, *Lactococcus*, *Lactobacillus*, *Pediococcus*, *Leuconostoc*), have been explored to a lesser extent than Gram‐negative bacteria, these probiotic microorganisms have garnered renewed interest as cancer therapies due to the extensive genetic toolbox existing for these bacteria and their safety and beneficial status within the human body (Bron & Kleerebezem, 2018). For example, an engineered *Lactococcus lactis* strain expressing a recombinant tumor metastasis‐inhibiting KiSS peptide was shown to inhibit the proliferation of human colonic epithelial cancer cells (Zhang et al., 2016). A variety of mechanisms can be employed on bacteria to achieve tumor therapy, including not only the innate toxicity of some bacteria, but also other features that can be incorporated into the bacterium of choice using recombinant DNA technology. For example, a bacterium can be engineered to produce an immunotherapy constituent that induces specific types of host immune cells or specific molecules that recognize and destroy cancer cells (such as cytokines, T‐cell lymphocytes, and tumor necrosis factors) (Leschner & Weiss, 2010). As another example, a bacterium could be engineered to produce specific enzymes (i.e., deaminases and nitroreductases) or bacteriocins that display cancer cell‐specific toxicities (Mills et al., 2022). Actually, there are many ways in which bacteria can be genetically improved to treat cancer and effectively destroy several types of tumors, such as increasing colonization of the tumor, improving targeting, or enhancing the release of therapeutics from bacteria, among other possibilities (Chien et al., 2017). One interesting biotherapeutic option for cancer treatment is the use of engineered bacteria to produce specific small interfering RNAs (siRNA) that target specific oncogenes. RNA‐interference (RNAi) is a potent mechanism of gene expression regulation, conserved from plants to humans, which holds promise for gene‐targeted therapies by specific silencing the genes of interest (Allahyari et al., 2023). Although the mechanism of RNAi does not occur in bacteria, it has been demonstrated that systemic gene silencing can be achieved in the nematode *Caenorhabditis elegans* when the animal ingests an *E. coli* engineered strain with the ability to produce a specific siRNA (Timmons & Fire, 1998). This result demonstrated that RNAi‐mediated information transfer between species or kingdoms was possible: a phenomenon that was termed as trans‐kingdom RNAi (tkRNAi). This phenomenon offers a practical solution for the in vivo application of RNAi in animals since the synthesis costs of artificial siRNAs suppose an extra limiting factor and its delivery is a handicap (May & Plasterk, 2005). One of the first researchers to apply this phenomenon of tkRNAi in gene silencing of oncogenes was Xiang and co‐workers (Xiang et al., 2006). These authors demonstrated that upon oral or intravenous administration of an engineered *E. coli* strain (encoding a siRNAs against catenin b‐1) were able to induce significant gene silencing of the targeted gene in human colon cancer xenografts in mice. These results provided an example of tkRNAi in higher organisms and highlighted the potential of bacteria‐mediated RNAi for development of clinically compatible RNAi‐based therapies. Since RNAi technology shows a high specificity towards mRNA binding, allowing differentiation between mRNAs with only one genetic point mutation (Acunzo et al., 2017; Ohnishi et al., 2008), this technology seems very appropriated and promising for the treatment of diseases caused by single point mutations of gain of function, like for example the conserved and distributed BRAF V600E (T1799A) mutation. This gain of function mutation has been thoroughly described in different types of cancer, including leukemia, melanoma, colorectal cancer, thyroid cancer, lung cancer and several types of gliomas, especially in pleomorphic xanthoastrocytoma (Di Nunno et al., 2023). Thus, in overall, live‐therapeutic bacteria (Figure 3) constitute a very promising alternative approach to treating a broad array of diseases, including cancer, and might reduce the problems derived from prolonged treatment generated with the classical systemically administered therapies, such as reduced efficacy and toxicity (Afrin et al., 2023).

### 5.4 Fecal microbiota transplantation

Live therapeutic products (Figure 3), constitute a novel class of therapeutics designed to prevent various recurrent bacterial infections in adults. Some of these live therapeutic products, such as those employed in the treatment of recurrent *Clostridium difficile* infection (CDI), have already received approval from several regulatory agencies, including the FDA (Gonzales‐Luna et al., 2023a). CDI arises following the invasion of toxins produced by the *C. difficile* bacterium, leading to symptoms such as diarrhea, abdominal pain, and in certain cases, toxic megacolon (Smits et al., 2016). Typically, the gut microbiota offers resistance to *C. difficile* colonization. However, alterations in this bacterial ecosystem due to various factors can facilitate the development of CDI. Complete recovery from CDI necessitates not only antibiotic treatment but also the restoration of a healthy balance within the host's microbiota (Gonzales‐Luna et al., 2023b). Presently, fecal microbiota transplantation (FMT) is the standard treatment for patients with multiple recurrent *C. difficile* infections. In the near future, the FDA and other regulatory agencies are expected to approve new commercially developed therapies derived from human microbiota. These therapies aim to treat diseases beyond CDI, such as inflammatory bowel disease and metabolic syndrome associated with type 2 diabetes (Gonzales‐Luna et al., 2023a).

## 6 CRISPR‐CAS SYSTEMS: A PRECISION GENOME EDITING TOOL FOR MEDICINE

The CRISPR Cas systems offer a novel approach to both RNA interference and genome editing (Mahato et al., 2024). When genetically engineered, CRISPR‐Cas systems serve as a valuable tool for controlling gene expression in both prokaryotic and eukaryotic cells. Approximately 90% of archaea and 40% of bacteria possess CRISPR‐Cas systems to provide resistance to foreign DNA elements (Barrangou et al., 2007). Some of these systems have been adapted as genetic engineering tools for medically important human cell lines (Mahato et al., 2024). In particular, the Class 2 Type II CRISPR‐Cas9 system from *Streptococcus pyogenes* has been utilized for a variety of genetic purposes in a wide range of organisms, from bacteria to humans (Cho et al., 2013). The wild‐type system contains a nuclease protein (Cas9) that cuts specific DNA sequences based on a single guide RNA (sgRNA) that specifically interacts with the Cas9 proteins and the DNA sequence it targets. Conversely, nuclease‐deactivated CRISPR–Cas (dCas) systems, which do not cut DNA, can be used to modulate transcription in cells and organisms (Qi et al., 2013). An alternative approach to bypass the limitations of double‐stranded break DNA knock‐out‐based protocols is to induce mRNA knockdown at the target site (i.e., silencing the expression of the target gene instead of knocking out the gene). Several Cas12 and Cas13 proteins have been shown to possess RNA‐guided RNase activity in a manner analogous to RNA‐guided DNA targeting CRISPR‐Cas9 effectors (Nie et al., 2023). Performing mRNA knockdowns instead of gene knockouts (in which the gene is irreversibly deleted) is very useful for genes that cannot be completely removed because they are essential for cell viability. In any case, because the CRISPR‐Cas platform is concise and self‐contained (and the sgRNA can be easily customized), it has the potential to be adapted for different purposes, such as performing gene knockouts, knock‐ins, the addition of tags, or controlling the expression of a gene of interest (Barrangou et al., 2007). The emergence of CRISPR‐Cas technology in the biomedical field marked an important milestone in the challenging goal of finding targeted therapies for complex diseases such as cancer or other genetic disorders (Mahato et al., 2024). A further advancement in the CRISPR‐Cas systems is the use of CRISPR‐associated transposase systems (CAST) found in certain specific microorganisms, such as certain cyanobacteria. For example, the natural CAST system from cyanobacteria *Scytonema hofmanni* (ShCAST) consists of Tn7‐like transposase subunits (TnsB, TnsC, and TniQ), a CRISPR array trans‐activating RNA, and a Cas12k protein. ShCAST catalyzes RNA‐guided DNA transposition by unidirectionally inserting segments of DNA 60 bp downstream of the protospacer adjacent motif (PAM) (Strecker et al., 2019). The PAM motif is a specific and variable (depending on the respective CRISPR‐Cas system) 2–6 bp DNA sequence immediately following the DNA sequence targeted by the sgRNA‐Cas nuclease complex (Barrangou et al., 2007). When the natural ShCAST system was heterologously expressed in *E. coli*, the genetically developed system was shown to integrate DNA into specific targeted sites of the *E. coli* genome with frequencies of up to 80% (without positive selection markers or additional requirements such as DNA repairing templates) (Strecker et al., 2019). Therefore, the CAST system constitutes an excellent tool for performing precision DNA insertions (“knock‐ins”) in targeted genes with few additional requirements. In summary, CRISPR‐Cas and its derivative systems have emerged as revolutionary platforms in medicine, providing an unparalleled, finely‐tuned gene editing tool for both DNA and RNA targeting, functional genomics, gene therapy, disease modeling, and cancer research, among other applications (Zhou et al., 2023). The key applications of CRISPR‐Cas systems in medicine are outlined below.

### 6.1 Gene editing and therapeutics

CRISPR‐Cas systems enable precise genome modification by targeting specific DNA sequences. By rectifying disease‐causing mutations or introducing therapeutic genes, CRISPR‐Cas‐based gene therapies present promising pathways for developing effective treatments for genetic diseases such as cystic fibrosis, sickle cell disease, and Duchenne muscular dystrophy. Beyond genome editing, CRISPR‐Cas systems can also be employed for programmable epigenetic modifications, facilitating targeted DNA methylation or demethylation (Yahsi et al., 2024). Furthermore, using CRISPR–Cas‐based transactivation approaches (CRISPRa) with dCas proteins or antibody‐mediated recruitments, transcriptional activators can be recruited to genomic regulatory elements, enabling potent and versatile synthetic transcriptional control on genes of interest, such as dysregulated genes causing disease (Mahata et al., 2023). Overall, these capabilities of the CRISPR‐Cas systems hold immense promise for advancing precision medicine initiatives, allowing the development of personalized therapies tailored to individual patients' genetic profiles.

### 6.2 Cancer research, infectious disease control, and disease modeling

CRISPR‐Cas technology facilitates the study of tumor suppressor genes and oncogenes by modulating gene expression and allowing the construction of either knock‐out or knock‐in mutants. The resulting knowledge can be utilized to develop targeted cancer therapies and identify biomarkers for early detection and prognosis. CRISPR‐Cas systems also enable the creation of cellular and animal models that closely mimic human diseases. Researchers can introduce disease‐associated mutations into cells or organisms to study disease mechanisms, screen potential drugs, and develop personalized therapies (Lopes & Prasad, 2024). In addition, CRISPR‐based screening platforms enable high‐throughput interrogation of gene function across the entire genome, allowing researchers to identify genes involved in disease pathways, drug resistance, and therapeutic responses. Lastly, CRISPR‐Cas systems offer innovative strategies for combating infectious diseases. These systems can be programmed to destroy viral and bacterial genomes, offering alternative treatments to antivirals and antibiotics (Zahedipour et al., 2024).

## 7 BIOSENSORS USING WHOLE BACTERIAL CELLS

Modern medicine necessitates the rapid identification of pathogenic microbial strains to choose the best possible treatment as quickly as possible. In this regard, nanotechnology enables the development of highly sensitive and specific biosensors to detect biomarkers, greatly expanding diagnostic possibilities. A biosensor is an instrument used to detect and/or measure a specific compound (i.e., “analyte”), which incorporates a biological agent to act as a “recognition agent” for the “analyte” that will be detected or measured. All biosensors consist of a detector (the component that interacts with the analyte) and a transducer (which transforms the detected interaction into a measurable analytical signal). There are different kinds of biosensors depending on the type of recognition agent in the detector, which can vary from an entire bacterial cell to a very specific component (such as an enzyme, an antibody, or an aptamer) (Li et al., 2024). Aptamers are single‐stranded oligonucleotides with sizes between 70 and 100 nucleotides that form specific three‐dimensional structures and are capable of recognizing various types of target molecules with high affinity (similar to antibodies). Aptamers are expected to provide superior affinity to antibodies since they can be selected and enhanced with in vitro techniques using a screening method known as SELEX (Uğurlu et al., 2024). Therefore, these molecules are being investigated as a real alternative to monoclonal antibodies in biomedical research. However, for some applications of certain biosensors, an entire cell is employed rather than a specific component, for example, an antibody. The use of bacteria as recognition agents in the detectors of biosensors offers a low‐cost, compact, and robust option for the continuous detection of analytes in the context of their application. In this sense, optimal designs for the integration of biosensors into final medical contexts, such as ingestible capsules, will be crucial to ensure their efficacy and safe use in clinical therapy (Chiang & Hasty, 2023; Hoang Trung Chau et al., 2020).

## 8 BACTERIOPHAGE‐BASED THERAPIES TO COMBAT FORMS OF RESISTANCE IN BACTERIA

**Figure 4:**
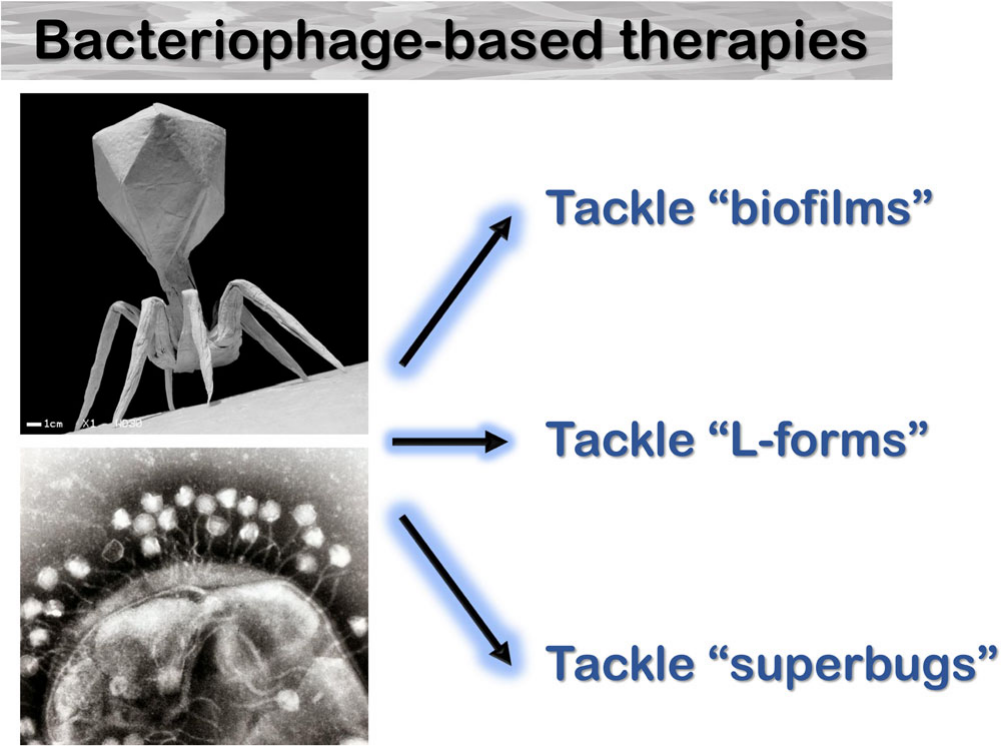
Bacteriophage‐based therapies to combat forms of resistance in bacteria. Bacteriophages, viruses that infect bacterial cells, are emerging as promising tools to address challenging infections. These include those stemming from “superbugs” (multidrug‐resistant bacteria), “biofilms” (communities of bacteria shielded by polysaccharides), and “L‐forms” (cell wall‐deficient bacteria).

Bacterial spores, including exospores and endospores, represent the most prominent forms of bacterial resistance, particularly endospores. These spores pose a risk when generated in hospitals, where they can survive most of the disinfectant compounds typically used (Dyer et al., 2019). Endospores produced by Bacillus and Clostridium species are extremely resistant to intense heat and irradiation. In addition to endospores, bacteria have evolved other natural mechanisms that protect them from antibiotics, disinfectants, and natural host defenses. These include the production of capsules, biofilms, and the so‐called L‐forms. Capsules are nonmetabolically active structures composed of repetitive units of polysaccharides or polypeptides. They provide important properties to bacterial cells, including adhesion to sister cells (forming colonies) and adhesion to inert or living substrates (forming biofilms) (Hall‐Stoodley et al., 2004). Growth in biofilms represents the typical way bacteria grow in nature. However, when formed on inert surfaces (such as catheters and surgical prostheses) or living substrates (such as tissues of higher organisms), these structures protect bacteria from host immune responses and antibiotic treatment. Therefore, biofilm‐associated infections can have a serious impact on health, making the diagnosis and treatment of biofilms challenging (Pai et al., 2023). One of the most promising therapeutic alternatives to antibiotics for treating these forms of bacterial resistance is bacteriophage‐based therapy (Usman et al., 2023). Bacteriophages (Figure 4) are viruses that exclusively infect bacteria and are among the most numerous, diverse, and dynamic biological entities on this planet (Fogg et al., 2017). Bacteriophages possess a remarkable ability to infect and destroy bacteria with precision, and they can be specifically customized to attack particular strains or species, including problematic encapsulated bacteria; in fact, capsules serve as receptors for many bacteriophages (Carascal et al., 2022). Several studies have shown that bacteriophage‐based therapy could be an effective treatment for biofilm infections affecting the skin, the gastrointestinal tract, and the urinary tract, among other tissues (Saeed et al., 2023). Bacteriophage‐based therapy could also be highly beneficial in combating chronic diseases due to L‐form bacterial infections, a term used to denote cell wall‐deficient bacteria (Errington et al., 2016). The peptidoglycan cell wall is highly conserved across the domain of bacteria, suggesting its early appearance in bacterial evolution (Errington, 2013). While normally essential, under certain stress conditions, wall‐deficient (or “L‐form”) bacteria can be isolated. The cell wall is the target for most commonly used antibiotics, and therefore, L‐forms are completely resistant to antibiotics that specifically act on cell wall synthesis, such as penicillins and cephalosporins (Errington, 2017). In many cases, the host's immune system recognizes epitopes associated with the cell wall, so the absence of this layer allows bacteria to evade their host's defenses, linking L‐forms to the development of chronic diseases. Furthermore, wall‐deficient cells are flexible, able to squeeze through narrow spaces, and capture exogenous DNA, which increases the risk of infections with these bacterial forms (Claessen & Errington, 2019). Therefore, new therapeutic strategies, such as bacteriophage‐based therapy, are still needed to address the real consequences that L‐forms have in both the containment of bacteria in certain tissues and their resistance to antibiotics and host immune defenses. However, there are still several obstacles to overcome in the production and safety of bacteriophages for the clinical use of this type of therapy (Saeed et al., 2023).

## 9 BIOPLASTICS OF MICROBIAL ORIGIN FOR DEVELOPING MEDICAL DEVICES

Plastics have revolutionized human lives, finding a wide range of applications from packaging to medical devices (García‐Depraect et al., 2021). The introduction of bioplastics to the market has opened a new perspective in the field of medicine. Due to their bioresorbability, these bioplastics can pass through biological barriers and concentrate in specific tissues (Silva et al., 2023). Among all existing bioplastics, only polyhydroxyalkanoates (PHAs) are fully synthesized by prokaryotic microorganisms (Bordel et al., 2021). Owing to their excellent biodegradability and biocompatibility properties, PHAs have emerged as one of the most promising biomaterials for the development of medical devices, including applications in tissue engineering, drug delivery, and orthopedics. Among the different PHAs, poly(3‐hydroxybutyrate‐co‐3‐hydroxyvalerate) (PHBV) has garnered significant interest in the field of biomedicine. The encapsulation of various drugs (i.e., anticancer, antibiotic, and anti‐inflammatory compounds) in PHBV microspheres is currently being extensively investigated. Overall, PHBV is a highly intriguing biomaterial for drug delivery, as it exhibits prolonged release kinetics with high drug stability, significantly reduced side effects, and greater bioavailability compared to other materials (Rodríguez‐Cendal et al., 2023). Additionally, PHBV has been tested as a support material for tissue engineering applications, such as bone, cartilage, and skin regeneration. The incorporation of PHBV into scaffolds appears to improve mechanical properties, biocompatibility, and cellular interactions, making them suitable for tissue engineering constructs (Wei & Tan, 2023). However, PHAs are not yet among the biomaterials approved by the FDA for use in clinics. Indeed, the two main bioplastics used as devices for drug delivery systems approved by the FDA are polylactic acid (PLA) and poly‐lactic‐co‐glycolic acid (PLGA). In particular, the use of PLGA nanoparticles as drug‐delivery systems to treat brain diseases has been widely investigated in recent years (Lamparelli et al., 2023). For example, two different preclinical studies have obtained promising results with PLGA nanoparticles loaded with either doxorubicin or chlorotoxin‐conjugated morusin for the chemotherapy of glioblastoma (Agarwal et al., 2019; Maksimenko et al., 2019). Nonetheless, PHAs are driving the growth of the biodegradable bioplastics market, and the production capacity is expected to triple in the next 5 years. Therefore, it is anticipated that similar to PLA and PLGA, natural PHA biopolymers will soon find various applications in the biomedical field, offering innovative approaches to numerous treatments and contributing to the advancement of modern medicine (Bhatia et al., 2021; Silva et al., 2023).

## 10 CONCLUSION

Advances in genetic engineering and microbial genetics are crucial for the development of various biotechnology processes, which are currently categorized as red (medical processes), white (industrial processes), yellow (food production processes), and green (agricultural processes) biotechnologies. Consequently, it is possible to address the challenges of today's society through research and innovation focused on the simplest forms of life, namely, microorganisms. Microbial biotechnology is the discipline most frequently employed by biotechnologists, microbiologists, biochemists, environmentalists, and genetic engineers. However, a significant amount of research is still needed to spark a true biotechnological revolution in most of society's productive sectors, including the pharmaceutical and medical fields. The findings presented in this review serve as an example of what scientists and clinicians can contribute to medicine. By working together, we can navigate the complex landscape of human disease, enhance global health, and make diseases curable.

## AUTHOR CONTRIBUTIONS

**Fernando Santos‐Beneit**: Conceptualization; writing—original draft; writing—review & editing.

## CONFLICT OF INTEREST STATEMENT

None declared.

## ETHICS STATEMENT

None required.

## 1

**Table A1:** Major antibiotics in clinical use approved by key regulatory agencies.

Antibiotic	Class	Mode of action (target)	Origin and producer
Amikacin	Aminoglycoside	Inhibitor of protein synthesis	Semisynthetic
Amoxicillin	β‐lactam (penicillin)	Inhibitor of cell wall synthesis	Semisynthetic
Ampicillin	β‐lactam (penicillin)	Inhibitor of cell wall synthesis	Semisynthetic
Azidamfenicol	Amphenicol	Inhibitor of protein synthesis	Semisynthetic
Azithromycin	Macrolide	Inhibitor of protein synthesis	Semisynthetic
Aztreonam	β‐lactam (monobactam)	Inhibitor of cell wall synthesis	Semisynthetic
Bacitracin	Polypeptide	Disruption of cell membrane and cell wall integrity	Natural (*Bacillus licheniformis*)
Bedaquiline	Diarylquinoline	Inhibitor of cellular energy production	Synthetic
Benzylpenicillin (penicillin G)	β‐lactam (penicillin)	Inhibitor of cell wall synthesis	Natural (*Penicillium chrysogenum*)
Cefadroxil	β‐lactam (cephalosporin)	Inhibitor of cell wall synthesis	Semisynthetic
Cefazolin	β‐lactam (cephalosporin)	Inhibitor of cell wall synthesis	Semisynthetic
Cefepime	β‐lactam (cephalosporin)	Inhibitor of cell wall synthesis	Semisynthetic
Cefiderocol	β‐lactam (cephalosporin)	Inhibitor of cell wall synthesis	Semisynthetic
Cefixime	β‐lactam (cephalosporin)	Inhibitor of cell wall synthesis	Semisynthetic
Cefminox	β‐lactam (cephalosporin)	Inhibitor of cell wall synthesis	Semisynthetic
Cefotaxime	β‐lactam (cephalosporin)	Inhibitor of cell wall synthesis	Semisynthetic
Ceftaroline	β‐lactam (cephalosporin)	Inhibitor of cell wall synthesis	Semisynthetic
Ceftazidime	β‐lactam (cephalosporin)	Inhibitor of cell wall synthesis	Semisynthetic
Ceftobiprole	β‐lactam (cephalosporin)	Inhibitor of cell wall synthesis	Semisynthetic
Ceftolozane	β‐lactam (cephalosporin)	Inhibitor of cell wall synthesis	Semisynthetic
Ceftriaxone	β‐lactam (cephalosporin)	Inhibitor of cell wall synthesis	Semisynthetic
Cefuroxime	β‐lactam (cephalosporin)	Inhibitor of cell wall synthesis	Semisynthetic
Ciprofloxacin	Quinolone	Inhibitor of nucleic acid synthesis	Synthetic
Clarithromycin	Macrolide	Inhibitor of protein synthesis	Semisynthetic
Clindamycin	Lincosamide	Inhibitor of protein synthesis	Semisynthetic
Clofazimine	Riminophenazine	Inhibitor of nucleic acid synthesis	Synthetic
Cloxacillin	β‐lactam (penicillin)	Inhibitor of cell wall synthesis	Semisynthetic
Colistin (polymyxin E)	Polypeptide	Disruption of cell membrane integrity	Natural (*Paenibacillus polymyxa*)
Dalfopristin	Streptogramin	Inhibitor of protein synthesis	Semisynthetic
Dapsone	Sulfone	Inhibitor of nucleic acid synthesis	Synthetic
Daptomycin	Lipopeptide	Disruption of cell membrane integrity	Natural (*Streptomyces roseosporus*)
Delafloxacin	Quinolone	Inhibitor of nucleic acid synthesis	Synthetic
Dicloxacillin	β‐lactam (penicillin)	Inhibitor of cell wall synthesis	Semisynthetic
Doripenem	β‐lactam (carbapenem)	Inhibitor of cell wall synthesis	Semisynthetic
Doxycycline	Tetracycline	Inhibitor of protein synthesis	Semisynthetic
Ertapenem	β‐lactam (carbapenem)	Inhibitor of cell wall synthesis	Synthetic
Erythromycin	Macrolide	Inhibitor of protein synthesis	Natural (*Saccharopolyspora erythraea*)
Fidaxomicin	Macrolide	Inhibitor of protein synthesis	Natural (*Dactylosporangium auranticum*); also produced by other actinomycetes
Fosfomicyn	Phosphonate	Inhibitor of cell wall synthesis	Synthetic^a^
Gentamicin	Aminoglycoside	Inhibitor of protein synthesis	Natural (*Micromonospora purpurea*)
Imipenem	β‐lactam (carbapenem)	Inhibitor of cell wall synthesis	Semisynthetic
Levofloxacin	Quinolone	Inhibitor of nucleic acid synthesis	Synthetic
Linezolid	Oxazolidinone	Inhibitor of protein synthesis	Synthetic
Mecillinam	β‐lactam (penicillin)	Inhibitor of cell wall synthesis	Semisynthetic
Meropenem	β‐lactam (carbapenem)	Inhibitor of cell wall synthesis	Semisynthetic
Metronidazole	Metronidazole	Inhibitor of nucleic acid synthesis	Synthetic
Minocycline	Tetracycline	Inhibitor of protein synthesis	Semisynthetic
Moxifloxacin	Quinolone	Inhibitor of nucleic acid synthesis	Synthetic
Nafcillin	β‐lactam (penicillin)	Inhibitor of cell wall synthesis	Semisynthetic
Netilmicin	Aminoglycoside	Inhibitor of protein synthesis	Semisynthetic
Norfloxacin	Quinolone	Inhibitor of nucleic acid synthesis	Synthetic
Ofloxacin	Quinolone	Inhibitor of nucleic acid synthesis	Synthetic
Oxacillin	β‐lactam (penicillin)	Inhibitor of cell wall synthesis	Semisynthetic
Phenoxymethylpenicillin (penicillin V)	β‐lactam (penicillin)	Inhibitor of cell wall synthesis	Natural (*Penicillium chrysogenum*)
Piperacillin	β‐lactam (penicillin)	Inhibitor of cell wall synthesis	Semisynthetic
Polymyxin B	Polypeptide	Disruption of cell membrane integrity	Natural (*Paenibacillus polymyxa*)
Quinupristin	Streptogramin	Inhibitor of protein synthesis	Semisynthetic
Rifampicin (rifampin)	Ansamycin	Inhibitor of nucleic acid synthesis	Semisynthetic
Roxithromycin	Macrolide	Inhibitor of protein synthesis	Semisynthetic
Sulfamethoxazole	Sulfonamide	Inhibitor of nucleic acid synthesis	Synthetic
Tedizolid	Oxazolidinone	Inhibitor of protein synthesis	Synthetic
Teicoplanin	Glycopeptide	Inhibitor of cell wall synthesis	Natural (*Actinoplanes teichomyceticus*)
Telithromycin	Ketolide	Inhibitor of protein synthesis	Semisynthetic
Temocillin	β‐lactam (penicillin)	Inhibitor of cell wall synthesis	Semisynthetic
Tetracycline	Tetracycline	Inhibitor of protein synthesis	Natural (*Streptomyces rimosus* and other spp.)
Thiamphenicol	Amphenicol	Inhibitor of protein synthesis	Semisynthetic
Tigecycline	Glycylcline	Inhibitor of protein synthesis	Semisynthetic
Tobramycin	Aminoglycoside	Inhibitor of protein synthesis	Semisynthetic
Trimethoprim	Diaminopyrimidine	Inhibitor of nucleic acid synthesis	Synthetic
Vancomycin	Glycopeptide	Inhibitor of cell wall synthesis	Natural (*Amycolatopsis orientalis*)

^a^ Fosfomycin was first isolated from cultures of *Streptomyces* spp. but currently is produced using a synthetic process (Dijkmans et al., 2017).

**Table A2:** Vaccines developed against major bacterial pathogens.

Bacteria	Disease	Type of vaccine^a^	Name/company/year of approval^b^
*Bacillus anthracis*	Anthrax	Inactivated vaccine	AVA (BioThrax)/Bioport/1970
*Bordetella pertussis*	Pertussis	Subunit vaccine^c^	Daptacel^c^/Sanofi‐Pasteur/1991
*Clostridium tetani*	Tetanus	Toxoid	Not in use today^c^
*Corynebacterium diphtheriae*	Diphtheria	Toxoid	Not in use today^c^
*Coxiella burnetii*	Q fever	Inactivated vaccine	Q‐Vax/Seqirus UK Limited/1989
*Francisella tularensis*	Tularemia	Live‐attenuated vaccine	LVS/no licensed in USA or EU^d^
*Haemophilus influenza b*	Hib infection	Conjugate vaccine	ActHIB/Sanofi‐Pasteur/1987
*Mycobacterium tuberculosis*	Tuberculosis	Live‐attenuated vaccine	BCG vaccine/Pasteur‐Merieux/1974
*Neisseria meningitidis*	Meningitis	Conjugate vaccine	NeisVac‐C/Pfizer/1999
*Salmonella enterica subsp. typhi*	Typhoid fever	Inactivated vaccine	Typhim Vi/Sanofi‐Pasteur/1994
*Streptococcus pneumoniae*	Pneumonia	Conjugate vaccine	Prevnar/Pfizer/2000
*Vibrio cholerae*	Cholera	Inactivated vaccine	Dukoral/Crucell/1991
*Yersinia pestis*	Plague	Inactivated vaccine	Not in use today^e^

^a^ Murray et al. (2021).

^b^ Only one example is included in the table with the year of approval by the FDA. There are distinct commercialized vaccines depending on the pharmaceutical company, the production year, and the country of use.

^c^ DTaP vaccines can prevent diphtheria (D), tetanus (T), and pertussis (aP), also known as “whooping cough.” *Vaccine information statement from Centers for Disease Control and Prevention (CDC)*.

^d^ In the 1900s, several vaccines were developed against tularemia including the killed “Foshay” vaccine, subunit vaccines comprising *F. tularensis* protein(s) or lipoproteins(s) in an adjuvant formulation, and the *F. tularensis* live vaccine strain (LVS); none were licensed in the USA or European Union (Jia & Horwitz, 2018).

^e^ A licensed vaccine was available in the United States of America (USA) from 1911 until 1999, when the single manufacturer stopped making it.
